# Targeting reversible post-translational modifications with PROTACs: a focus on enzymes modifying protein lysine and arginine residues

**DOI:** 10.1080/14756366.2023.2254012

**Published:** 2023-09-04

**Authors:** Marta Pichlak, Tomasz Sobierajski, Katarzyna M. Błażewska, Edyta Gendaszewska-Darmach

**Affiliations:** a Institute of Molecular and Industrial Biotechnology, Lodz University of Technology, Łódź, Poland; b Institute of Organic Chemistry, Lodz University of Technology, Łódź, Poland

**Keywords:** PROTAC, posttranslational modifications, E3 ubiquitin ligases, proteasome degradation, collateral degradation, structure optimisation

## Abstract

PROTACs represent an emerging field in medicinal chemistry, which has already led to the development of compounds that reached clinical studies. Posttranslational modifications contribute to the complexity of proteomes, with 2846 disease-associated sites. PROTAC field is very advanced in targeting kinases, while its use for enzymes mediating posttranslational modifications of the basic amino acid residues, started to be developed recently. Therefore, we bring together this less popular class of PROTACs, targeting lysine acetyltransferases/deacetylases, lysine and arginine methyltransferases, ADP-ribosyltransferases, E3 ligases, and ubiquitin-specific proteases. We put special emphasis on structural aspects of PROTAC elements to facilitate the lengthy experimental endeavours directed towards developing PROTACs. We will cover the period from the inception of the field, 2017, to April 2023.

## Introduction

A protein’s nascent structure is insufficient to cover a variety of biological processes. The spectrum of protein structures and functionalities is expanded by the chemical modifications of a polypeptide chain after its translation. Thus, with alternative splicing, posttranslational modifications (PTMs) contribute to the genome-independent complexity of proteomes. In general, PTMs refer to various irreversible or reversible covalent processing events, including proteolytic cleavage or covalent attachment of chemical groups to the target amino acid residue such as phosphate, acetate, sulphate, etc. PTMs affect a protein’s molecular weight, charge, conformation, stability, and consequently, activity, stability, and localization^1^.

There are approximately 700 unique entries in the UniProt database reflecting different types of PTMs to date. Phosphorylation, acylation, alkylation, glycosylation, and oxidation are the most prevalent additive PTMs^2^. According to the dbPTM, one of the most comprehensive databases that provide functional and structural analyses for PTMs, there are more than 2 000 0000 experimentally verified PTM sites with 2846 disease-associated. The greatest information about disease features is related to protein phosphorylation, which can have up to 1892 substrate sites. Therefore, PTMs are crucial for biomarker studies and therapeutics development since accumulating evidence indicates that their aberrant status is frequently associated with a variety of human disorders, including malignancies, diabetes, coronary heart, and neurodegenerative diseases^3^.

For reversible PTMs, two major classes of enzymes that add and remove modifications determine the amount, site specificity, and pattern of particular PTMs. Kinases, ubiquitin ligases, and acetyltransferases are examples of writers, while phosphatases, deubiquitinases, and deacetylases of erasers. To date, various drugs targeting modifying writers or erasers enzymes including kinases, histone deacetylase, and histone methyltransferase have been approved for the treatment of disease^4^. The landscape of drug development has changed since targeted protein degradation was first introduced more than twenty years ago with the discovery of PROteolysis TArgeting Chimaeras (PROTACs)^5^. The clinical candidate drugs known as PROTACs have developed from cell-impermeable peptide-small molecule chimeras to orally bioavailable forms that destroy oncogenic proteins. There are about a dozen PROTACs being developed for clinical use worldwide. The most advanced PROTAC in clinical development, Arvinas’ ARV-471 has entered clinical phase III in participants with advanced breast cancer (ClinicalTrials.gov NCT05654623).

**Figure 1.**
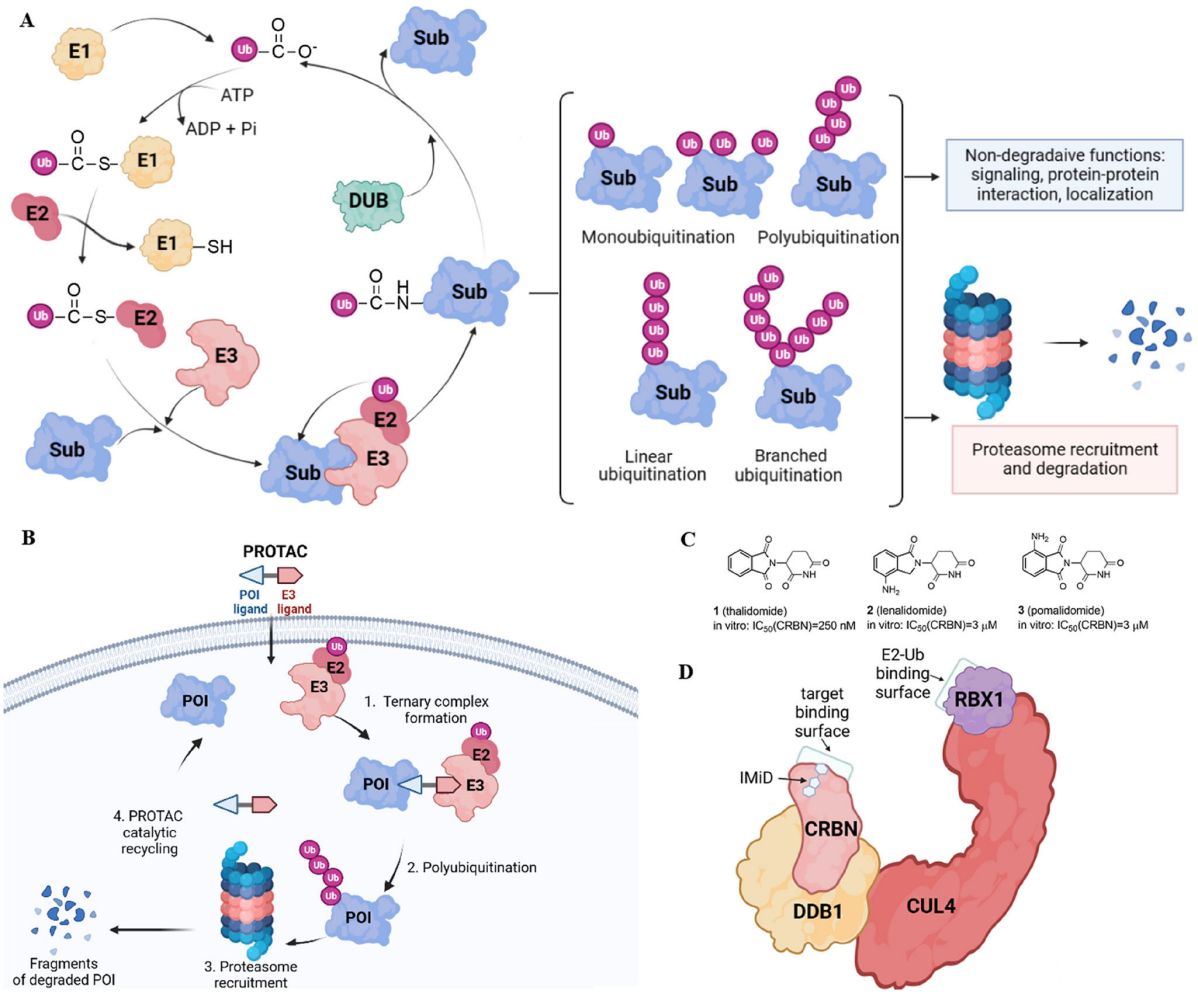
The ubiquitin-proteasome system is the basis for PROTACs’ mechanism (created in BioRender.com). Ubiquitination catalyzed by E1, E2, and E3 enzymes and deubiquitination by deubiquitination enzymes (DUBs). The proteasome breaks down the proteins that have been tagged with ubiquitin by specific enzymes (A); a POI ligand, an E3 ligand, and a linker are present in PROTAC. Inducing polyubiquitination and proteasome-mediated degradation of POIs is the function of the E3-PROTAC-POI ternary complex (B); chemical structures of immunomodulatory drugs approved by FDA (C) immunomodulatory drugs bind CRBN, a part of CUL4-RBX1-DDB1-CRBN complex (D).

The PROTAC approach is based on the ubiquitin-proteasome system (UPS) (Figure 1(A)). In eukaryotic cells, UPS is the primary pathway for the degradation of intracellular proteins. Ubiquitination is crucial for protein substrate degradation and, as a result, controls the “quantity” and “quality” of different proteins, ensuring cellular homeostasis. Ubiquitin (Ub), a 76-amino acid regulatory protein, can be covalently tagged to target proteins via a series of enzymatic events involving Ub-activating (E1), Ub-conjugating (E2), and Ub-ligating (E3) enzymes. E1 activates Ub in an ATP-dependent manner before transferring to E2. The Cys residue of E1 and the C-terminal carboxyl group of Ub form a thioester bond. In the second phase, E1 transfers activated Ub to E2 and supports E3 ligases in transferring active Ub to the substrate. Finally, E3 ligases are responsible for the transfer of Ub from E2∼Ub to a specific substrate protein. When this process is completed, an isopeptide bond is formed between the substrate’ lysine ε-amino group and the C-terminal carboxyl group of Ub. Three major types of ubiquitination have been discovered based on structural characteristics: monoubiquitination, polyubiquitination, and branching ubiquitination^6^. The amide bond between Ub moieties can be created using any of the seven lysine residues (K6, K11, K27, K29, K33, K48, and K63) or the N-terminal methionine on Ub. However, targeted proteins are primarily degraded by proteasomes using K48-linked polyUb chains^7^. Deubiquitination enzymes can trim the conjugated Ub molecule away from the target protein (DUBs).

The human genome contains around 600 E3 ligases, with the RING (**r**eally **i**nteresting **n**ew **g**ene) family being the biggest. The largest superfamily within that family is the cullin-RING E3 ligases (CRLs) with the core four components: the cullin (CUL) protein, which acts as the scaffold, the RING finger protein, which binds to an E2 ubiquitin conjugating enzyme, the substrate receptor, which identifies the target protein, and the adaptor proteins, which connect the substrate receptor and cullin. Also, several human diseases, such as cancer, infectious diseases, and neurological disorders, have been linked to E3 ligases, but only one E3 ligase-targeting drug type called immunomodulatory drugs (IMiDs) has received FDA approval to date. Thalidomide (**1**)^8^, lenalidomide (**2**), and pomalidomide (**3**)^9^ (Figure 1(C)) that target the substrate receptor Cereblon within CUL4-RBX1-DDB1-CRBN(CRL4^CRBN^) E3 ligase (Figure 1(D)) induce ubiquitination and degradation of Ikaros (IKZF1) and Aiolos (IKZF3), which are lymphoid transcription factors. Lenalidomide also causes ubiquitylation and subsequent degradation of casein kinase 1α. Therefore, IMiDs are effective in treating hematological malignancies like multiple myeloma and myelodysplastic syndrome^10^.

PROTACs are molecules consisting of a POI ligand and an E3 ubiquitin ligase recruiting ligand joined together with a linker. Through the formation of a ternary complex, PROTACs cause the target protein and an E3 ligase to be in close proximity, leading to the ubiquitination of the POI. The E3 ligase can be hijacked by the PROTAC E3 ligase ligand, which will then label the protein of interest (POI) with ubiquitin (Figure 1(B)). CRLs are the main E3 ligases exploited so far in PROTAC strategy with the von Hippel-Lindau (VHL), cerebron (CRBN), inhibitors of apoptosis (IAPs), and mouse double minute 2 (MDM2) being explored the most.

In this review, we summarise the recent developments in the design of PROTACs which target enzymes mediating PTMs. Kinases constitute the majority of proteins degraded by PROTACs as most kinases have well-known and effective inhibitors or ligands that can be easily changed to join linkers and maintain enough binding affinity. Furthermore, kinases have a deep binding pocket that can promote PROTAC binding, resulting in an interaction between the enzyme and the E3 ligase, ubiquitination, and finally degradation of the kinase^11^. Since 2013^12^, numerous research groups have proposed converting potent kinase inhibitors into PROTAC drugs and have reported relevant and intriguing data using this new technique that destroys proteins by utilizing cellular machinery.

Unlike phosphorylation, PROTAC molecules targeting other PTMs were developed much later. The first PROTACs degrading lysine acetyltransferases^13^ and lysine deacetylases^14^^,^^15^ appeared only in 2018. The first ADP-ribosyltransferase-addressing PROTAC was reported in 2019^16^ while arginine methyltransferases^17^ and lysine methyltransferase-targeted PROTACs^18^ were described in the literature in 2020 and 2021, respectively.

In 2017 first PROTACs for the auto-induced degradation of E3 ubiquitin ligase was developed^19^. Interestingly, the above-mentioned modifications, in contrast to the phosphorylation taking place on hydroxylated amino acids, include the basic amino acid residues, lysine, and arginine. Therefore, in this review, we are going to focus on PROTACs targeting enzymes modifying protein lysine and arginine residues (lysine acetyltransferases/deacetylases, lysine and arginine methyltransferases, ADP-ribosyltransferases, and Ub-specific proteases). We cover the period from 2017 to April 2023. We put special emphasis on structural aspects of key PROTAC elements collected in the form of comparative Tables.

## Acetylation

### Lysine acetyltransferases and lysine deacetylases

**Figure 2.**
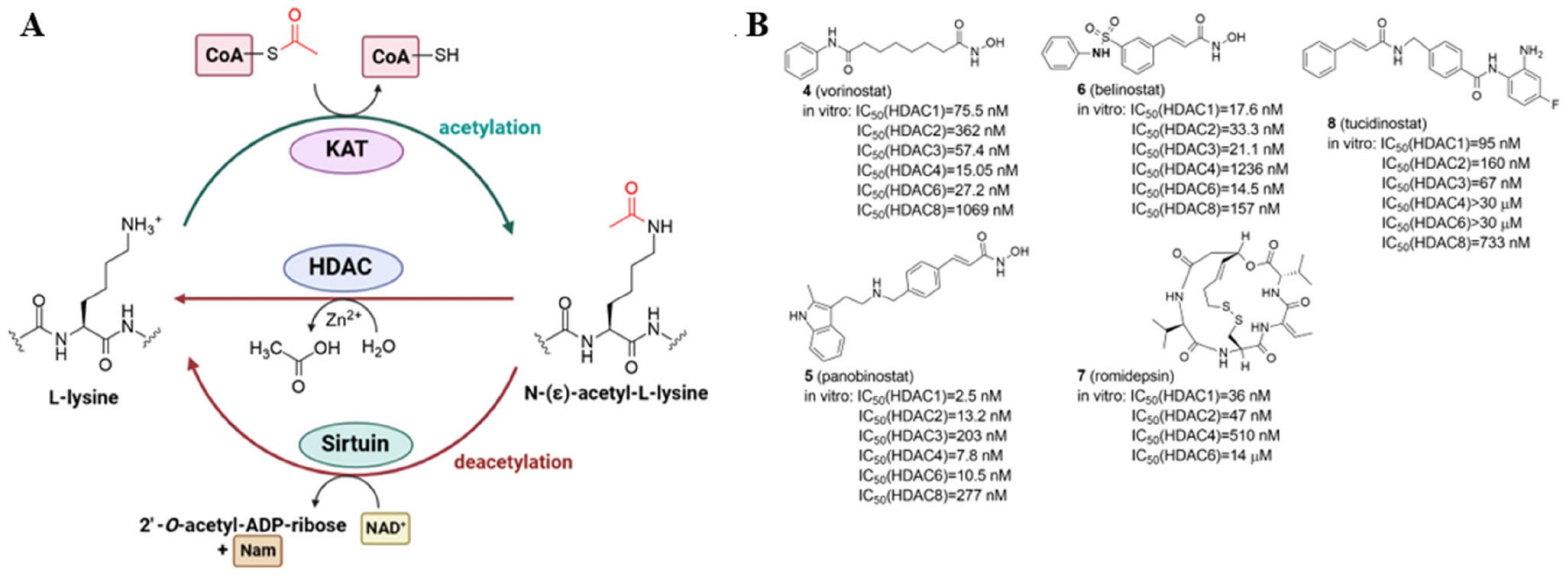
Protein lysine acetylation and deacetylation catalyzed by lysine acetyltransferases (KATs) and lysine deacetylases (KDACs) with Ac-CoA as the acetyl group donor (A); current HDAC inhibitors approved by FDA and China’s National Medical Products Administration (B).

Lysine acetylation, the process of transferring an acetyl group from acetyl-coenzyme A (Ac-CoA) to the primary amine in the ε-position of a protein’s lysine side chain, is a reversible PTM that changes the charge on lysine residues thereby affecting protein activity or stability. Although acetylation can occur nonenzymatically, the majority of known cases result from the balance of opposing enzymatic activities. Lysine acetylation is catalyzed by lysine acetyltransferases (KATs) while its deacetylation - by lysine deacetylases (KDACs) (Figure 2(A); Table 1). There are 18 KDACs expressed by two families in mammals, with the majority of them hydrolyzing the N-acetyl group from lysine residues on histones inside the nucleus. Additionally, several KDAC members can also target cellular non-histone proteins, such as structural proteins, chaperone proteins, transcriptional regulators, and DNA binding proteins. Specifically, HDAC1, 2, 3, and 8 belong to class I histone deacetylase (HDAC) located mainly in the nucleus, while HDAC8 is also found in the cytoplasm. Class IIa (HDAC4, HDAC5, HDAC7, HDAC9) have structural homology with class I and, except for HDAC9 localized in the nucleus, shuttle between the nucleus and the cytoplasm. Class IIb comprises HDAC10 which is structurally similar to class I and IIa and cytoplasmic HDAC6, the only HDAC family member with two tandem domains. Sirtuins (SIRT1–7), class III HDACs, and class IV (HDAC11) are functionally and structurally different from the other KDACs^20^. HDAC1–11 have a surface cavity with variable size and a narrow tunnel that connects to a Zn^2+^ ion in the active site while SIRTs in class III are NAD^+^-dependent^21^. The largest HDAC family member is HDAC6 with a zinc-finger ubiquitin-binding domain at the C-terminus and two independent catalytic domains (CD1 and CD2, located at the N-terminal and the central region, respectively). HDAC6 is largely responsible for the deacetylation of cytoplasmic proteins such as α-tubulin, heat shock protein 90, and cortactin, in contrast to other HDACs that mainly target histones in the nuclei^22^.

**Table 1.** Categorisation of KAT and KDAC enzymes.

Subfamily	Protein name	Aliases	Localisation	Subfamily	Protein name	Aliases	Localisation
KATs				KDACs			
	αTAT1		Cytoplasm, Nucleus	Class I	**HDAC1**	KDAC1	Nucleus
GNAT	KAT1	HAT1	Cytoplasm, Nucleus		**HDAC2**	KDAC2	Nucleus
	KAT2A	**GCN5** ^a^	Nucleus		**HDAC3**	KDAC3	Cytoplasm, Nucleus
	KAT2B	**PCAF**	Nucleus		**HDAC8**	KDAC8	Cytoplasm
	ATF2	CREB2	Nucleus, Mitochondria	Class IIa	**HDAC4**	KDAC4	Cytoplasm, Nucleus
P300/CBP	KAT3A	**CBP**	CytoplasmNucleus		HDAC5	KDAC5	Cytoplasm, Nucleus
	KAT3B	**P300**	CytoplasmNucleus		HDAC7	KDAC7	Cytoplasm, Nucleus Mitochondria
TAFII250	KAT4	TAF1	Nucleus		HDAC9	KDAC9	Cytoplasm, Nucleus
MYST	KAT5	TIP60	CytoplasmNucleus	Class IIb	**HDAC6**	KDAC6	Cytoplasm
	KAT6A	MYST3 MOZ	Nucleus		HDAC10	KDAC10	Cytoplasm, Nucleus
	KAT6B	MYST4 MORF	Nucleus	Class III	SIRT1		Cytoplasm, Nucleus
	KAT7	MYST2 HBO1	Nucleus		**SIRT2**		Cytoplasm, Nucleus
	KAT8	MYST1 MOF	Nucleus, Mitochondria		SIRT3		Nucleus, Mitochondria
ELP3	KAT9	ELP3	Cytoplasm, Nucleus		SIRT4		Mitochondria
	GCN5L1	BLOS1	Cytoplasm Mitochondria		SIRT5		Mitochondria
	KAT12	GTF3C4	Nucleus		SIRT6		Nucleus
SRCs	KAT13A	NCoA-1 SRC1	Cytoplasm, Nucleus		SIRT7		Nucleus
	KAT13B	NCoA-3 TRAM1	Cytoplasm, Nucleus Exosome	Class IV	HDAC11	KDAC11	Nucleus
	KAT13C	NCoA-2 TIF2	Cytoplasm, Nucleus				
	KAT13D	CLOCK	Cytoplasm, Nucleus				
	KAT14	CSR2B	Cytoplasm, Nucleus				

^a^Enzymes for which PROTACs have been developed are marked in bold

Isoenzyme-selective KDAC inhibitors are of interest as chemical tools and therapeutic medicines because each KDAC is considered to have distinctive substrate specificity connected with different diseases^23^. Since the HDAC family is frequently overexpressed in a variety of human cancers, HDAC inhibitors (HDACi) have been extensively tested as anticancer therapeutics. The four HDAC inhibitors with FDA approval include the hydroxamic acids vorinostat (**4**) (suberoylanilide hydroxamic acid, SAHA), panobinostat (**5**), and belinostat (**6**)^24^, and the cyclic peptide natural product romidepsin (**7**)^25^ (Figure 2(B)). Notably, the usage of HDAC inhibitors has shown to be primarily beneficial in hematological disorders. None of the FDA-approved HDACi have selectivity for a particular HDAC isoform because all of them contain a zinc-binding group (ZBG) and target the active site of the zinc-dependent I, II, and IV HDAC classes. The 2-aminoanilide tucidinostat (**8**)^26^, which underwent late-stage testing in Europe and the US, was the first class I and II-selective agent that was approved by China’s National Medical Products Administration in 2015^21^.

The lack of selectivity, and very modest efficacy, combined with dose-limiting toxicity and side effects of HDACi highlights the importance to develop new inhibitors or new approaches such as PROTACs. Also, resistance observed during HDACi treatment is a significant challenge^27^. Since all FDA-approved HDACi inhibit several HDAC isoforms, their restricted clinical toleration may be brought on by their promiscuous isoform profile^21^. Besides, although catalytic domains are blocked by conventional HDACi, HDAC can still affect cellular processes due to the interaction with many cellular proteins. Due to the elimination of the whole protein, targeted degradation of HDAC may be preferable to the well-known HDAC inhibition. What is more, it has been demonstrated that some PROTACs targeting HDAC can selectively degrade a specific isoform^28–32^.

### PROTACs targeting lysine acetyltransferases

**Figure 3.**
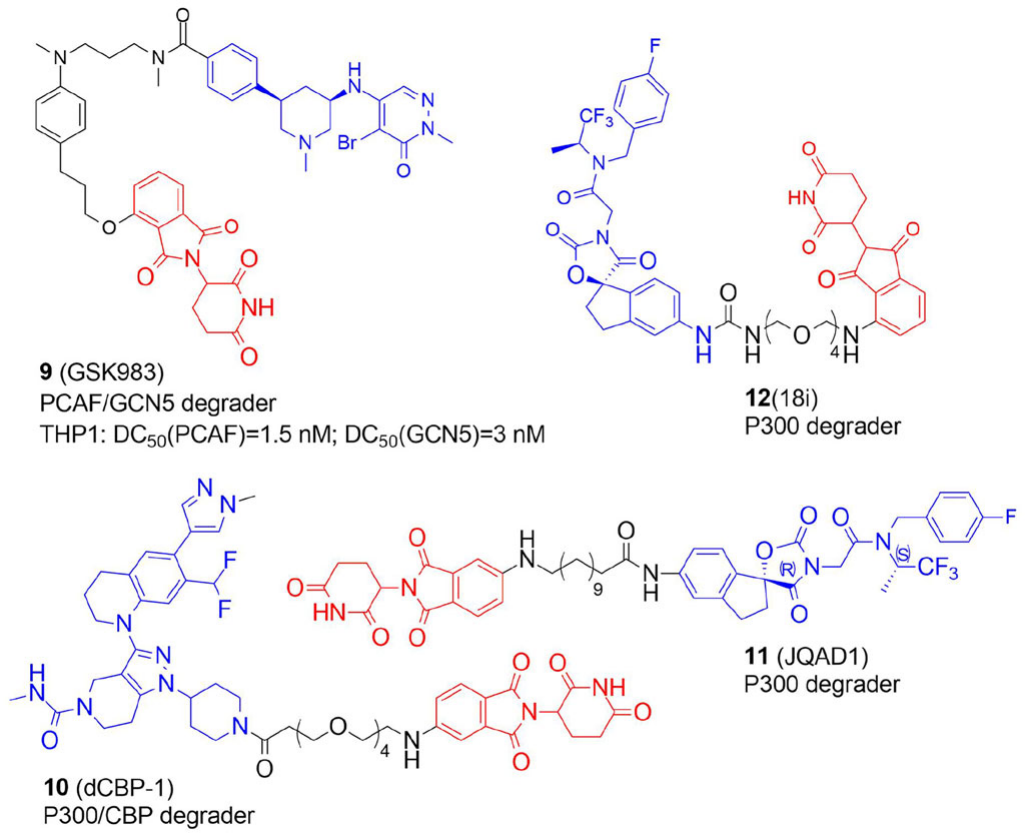
Structures and activities of PROTACs targeting KATs.

Unlike PROTACs targeting lysine deacetylases, a few examples of protein-degrading molecules with acetyltransferase activity have been published to date (Figure 3, Tables 2 and 3). There are 21 putative lysine acetyltransferases in the human proteome (Table 1). Based on structural and biochemical characteristics of catalysis as well as homology to yeast proteins, the best-characterized KATs have been grouped into three major families, GCN5-related N-acetyltransferases (GNAT), P300/CREB-binding protein (P300/CBP), and the MOZ, Ybf2, Sas2, and Tip60 (MYST) family. KATs play a crucial role in cellular physiology and pathology due to their broad substrate selectivity (including histones, transcriptional factors, kinases, and tumour suppressors) and participation in important cellular processes. Diseases including inflammatory illnesses, and pulmonary, cardiovascular, and neurological pathologies could all be brought on by the dysregulation of KAT expression or enzymatic activity^36^.

**Table 2.** Components and structural details of KAT-PROTACs.

PROTAC	Target	# of analogs	Original inhibitor	E3 ligase ligands	Type of linker (# of analogs)	Comments	Ref
**9** (GSK983)	PCAF/GCN5	3	GSK4027	CRBN	Alk (3)	*cis*-(*R*,*R*)-disubstituted piperidine analog more active than its *cis*-(*S*,*S*)-stereoisomer	^13^
**10** (dCBP-1)	P300/ CBP	1	GNE-781analog	CRBN	PEG (1)	**-**	^33^
**11** (JQAD1)	P300	2	(*R*,*S*)-A485	CRBN	Alk (2)	Computational modelling suggests that an ideal linker length between A485 and the CRBN would be a distance of 8–12 atoms; JQAD1 binds more avidly to P300 than CBP	^34^
**12** (18i)	P300	9	A485	CRBN	Alk (5) PEG (4)	Compounds with shorter linkers (<9 atoms) poorer degraders than those with longer; Conjugation through the aryl amine position of A-485 is suitable for P300 degradation	^35^

**Table 3.** Experimental methods and cell models applied in development of KAT-PROTACs.

PROTAC	Target	Cell lines	Cell proliferation/cell cycle/apoptosis	Target degradation	Other assays	Ref
**9** (GSK983)	PCAF/GCN5	THP-1, DCs, bone marrow cells, monocytes		Western blot	*In vitro:* Meso Scale Discovery; dot blots; qPCR arrays	^13^
**10** (dCBP-1)	P300, CBP	MM.1S, MM.1R, U266, H929, RPMI-8226, KMS-11, KMS-12-BM, KMS-12-PE, KMS20, KMS26, KMS27, KMS34, KMM1, OPM2, L363, MOLP-8, AMO-1, Karpus-620, SKMM2, EJM, LP1, MM.1S, KMS-12, KMS34,	Alamar Blue	Capillary-based immunoassays; mass spectrometry	*In vitro:* RNA-Seq; ChIP-seq; ATAC-seq	^33^
**11** (JQAD1)		BE2C, CHP212, IMR32, SKNSH, SHSY5Y, SKNAS, 293T, NB69, CHP134, Kelly, NGP, SIMA, MHHNB11, NBL-S, SHEP, NB5, NB1691, SKNMM, CHLA90, CCLF_PEDS_0046_N	crystal violet staining; CellTiter-Glo; flow cytometry	Western blot; mass spectrometry	*In vitro:* co-IP; RNA-seq; Biotin-JQAD1 pulldown; genome-wide occupancy analysis *In vivo*: CD1(ICR) mice (toxicity studies); CD1(ICR) and C57BL/6-*Crbn^tm1.1Ble^*/J, (MTD testing), NOD.Cg-*Prkdc^scid^* *Il2rg^tm1Wjl^*/SzJ (NSG) mice (tumour xenograft studies Kelly cells)	^34^
**12** (18i)	P300	MM.1S	MTS	Western blot		^35^

The first PROTAC targeting the KAT family was designed by Bassi et al. in 2018. to degrade P300/CBP-associated factor (PCAF) and general control nonderepressible 5 (GCN5), highly homologous multidomain proteins^13^. Numerous lysines in histones and other proteins, such as transcription factors and cytoskeletal components, are acetylated by PCAF and GCN5. PCAF is involved in the production of TNF and IL-6 cytokines and therefore might represent a potential therapeutic target for inflammatory diseases. Pharmacological inhibition of PCAF/GCN5 was insufficient but the small molecule GSK4027-based PROTAC with thalidomide GSK983 (**9**) induced degradation of PCAF/GCN5 with low nanomolar range and was shown to control the expression of inflammatory mediators in macrophages and dendritic cells. **9** was a mixture of diastereomers, with a *cis-*stereochemistry across the piperidine ring. The *cis*-(*R*,*R*)-isomer was also efficient in PCAF/GCN5 degradation while the *cis*-(*S*,*S*)-isomer was less active.

In 2021, the Ott group synthesized a highly potent and selective P300/CBP-targeting CRBN-recruiting PROTAC, dCBP-1 (**10**). The chromatin regulators P300/CBP serve as a promising therapeutic target for cancer, HIV infection, or metabolic diseases. Originally thought to be histone acetyltransferases, they are now shown to dynamically acetylate 21 000 lysines on more than 5000 different proteins. However, the relatively shallow substrate-binding site in P300 is a difficult pharmacological target, and up to now, most substances have focused on the enzyme’s acetyl-CoA binding site. Besides, inhibition of single domains cannot completely deactivate P300/CBP. **10** was designed by replacing the tetrahydropyran ring of P300/CBP bromodomain inhibitor GNE-781 with piperidine and linking it via 4 units of PEG with thalidomide^33^. The rapid and selective dual degradation of CBP and P300 demonstrated the versatility of using PROTAC technology to handle exceptionally large proteins (>300 kDa). In comparison to bromodomain and KAT domain inhibitor treatment, either alone or in combination, augmented effects on gene expression, antiproliferation, and chromatin structures of **10** activity were observed in multiple myeloma cells. With **10** treatment, both up- and down-regulated genes were identified, but the oncogene MYC was the most downregulated among all transcripts examined.

In a recent study, Durbin et al. created JQAD1 (**11**) by linking a potent and specific inhibitor towards P300/CBP (oxazolidinedione urea A485) with the CRBN ligand^34^. Both (*R*,*S*) and (*S*,*S*) stereoisomers of A485 were modified with the 12 C linker. The (*R*,*S*) diastereomer appeared to have the lowest IC_50_ value in three neuroblastoma cell lines. Importantly, the apparent selectivity of **11** for binding to P300, not CBP, was demonstrated and resulted in its proteasomal degradation and cell death linked to MYCN downregulation. This selectivity contrasts sharply with A485's more promiscuous P300 and CBP-specific acetyltransferase inhibitory activities. Because P300, not CBP, is the primary modulator of histone H3 lysine 27 acetylation (H3K27ac) in high-risk neuroblastoma, preferential targeting of P300 by this PROTAC is advantageous. **11** was also efficacious *in vivo* in a CRBN-dependent manner and exhibited minimal toxicity to untransformed cells. Rapid reduction of MYCN expression and loss of MYCN-driven transcriptional activity are the outcomes of P300 degradation.

By targeting P300/CBP, Brownsey et al. reported a new strategy for discovering optimal linkage sites based on biotinylated protein ligands^35^. A-485 inhibitor was chosen as a feasible starting point and coupled through urea moiety with CRBN ligand. A conjugation site through a urea moiety was identified and nine A-485-based PROTACs were synthesized. The most effective compound 18i (**12**) tested in cellular viability and P300 degradation assays in a myeloma cell line, MM.1S contained a PEG4 linker.

### PROTACs targeting lysine deacetylases: SIRT2

PROTACs targeting KDACs are a very new area of study (Tables 4 and 5). The first degraders of the SIRT2 were developed in 2018. Schiedel *et al*. developed a SIRT2 degrader using a triazole-based analog of selective SIRT2 inhibitor forcing a rearrangement of the active site upon ligand binding (SirReals, sirtuin rearranging ligands). PROTAC 12 (**13**) (Figure 3) was synthesized with a protocol based on a Cu(I)-catalyzed cycloaddition of an azido-thalidomide conjugate to the SirReal ligand. **13** efficiently inhibited the activity of SIRT2 in HeLa cells in an isotype-selective way resulting in hyperacetylation of the microtubule network^14^.

**Table 4.** Components and structural details of KDAC-PROTACs.

PROTAC	Target	# of analogs	Original inhibitor	E3 ligase ligands^a^	Type of linker (# of analogs)	Comments	Ref
**13** (12)	SIRT2	1	SirReal	CRBN	Alk (1)	–	^14^
**14** (TM-P4-Thal)	SIRT2	2	TM	CRBN	PEG (2)	The efficiency in the SIRT2 degradation was lower for PROTAC with shorter linker	^37^
**15** (PRO-SIRT2)	SIRT2	1	probe 3A	CRBN	Other (1)	–	^38^
**16** (4)	HDAC1/2/3	4	CI-994	**VHL** CRBN	Alk (4)	VHL-based more effective degrader than CRBN-based; longer linker (C12) is more effective in histone acetylation	^39^
**17** (JPS014) **18** (JPS016) **19** (JPS036)	HDAC1/2/3	24	CI-994	VHL	Alk (17) PEG (6) Other (1) (alk-piperazine)	An increase in HDAC1/2 degradation with increasing alkyl linker from C9 to C12 and C14; Incorporation of PEG or piperazine resulted in an almost complete loss of HDAC1/2 degradation; Incorporating one oxygen atom into a 12-atom linker (7) resulted in HDAC1/2 degradation; Incorporation of one oxygen atom into a 12-atom linker resulted in enhanced degradation of HDAC1 and HDAC3; Substitution of the acetyl group for a fluorinated cyclopropane ring in VHL led to an HDAC3-selective **15**	^40^
**20** (JPS026) **21** (JPS004)	HDAC 1/2/3	7	CI-994	VHL IAP	Alk (7)	the level of histone acetylation and the number and degree of differentially expressed genes increased with longer linkers (≥11 atoms); Short linkers: better HDAC inhibition *in vitro* but a decrease to nine atoms caused a loss of HDAC degradation; VHL- and IAP-based PROTACs degrade HDAC1/2/3 and induce histone acetylation to a similar degree; IAP-based PROTAC more cytotoxic	^41^
**22** (JMC-137)	HDAC1/2	11	MS-275 (Etinostat)	VHL	Alk-triazole-alk (10) Alk-triazole-PEG (1)	Dependence on linker length with 12 atoms exhibiting the most prominent HDAC1/2 degradation; Incorporating the carbamate and heterocycle groups of MS-275 in combination with shorter linkers (less than 12 atoms) did not increase potency; JMC-137 with the triazole group positioned further away from the carbamate moiety and HDAC ligand preferentially degrades HDAC1/2 over HDAC3	^42^
**23** (2)	HDAC1/2/3	4	trapoxin	CRBN	Alk-triazole (4)	Higher potency for compounds with longer tyrosine-based linker; degradation was more potent for the ethyl ketone-bearing than for hydroxamate PROTAC	^43^
**24** (XZ9002)	HDAC3	7	SR-3558	**VHL** CRBN	PEG (3) Alk (4)	In VHL-based PROTACs with alkyl linkers, the HDAC binding activities decreased with the linker length; VHL PROTACs more potent and selective in HDAC3 degradation	^44^
**25** (HD-TAC7)	HDAC3	7	CI-994	CRBN	Alk (3), alk-amide-alk (4)	Slightly higher activity of compounds with longer linkers with amide bond built-in; modification of *o-*aminoanilide with a fluorine improved HDAC3 selectivity degradation depends on the cell line (HDAC3 not degraded A549 cells in contrast to RAW 264.7);	^28^
**26** (7) **27** (11)	HDAC4	6	HA hydroxamic acid; TFMO-trifluoromethy loxadiazole	VHL	PEG-Alk (6)	Little difference in DC_50_ across the HA and TFMO series; PEG linker length does not influence degradation efficiency	^45^
**28** (9c)	HDAC6	4	2	CRBN	PEG (4)	Among the four linker lengths, **9c** with 3 PEGs showed the highest activity	^15^
**29** (NP8)	HDAC6	4	Nexturastat A	CRBN	PEG (4)	Among the four linker lengths, NP8 with 2 PEGs showed the highest activity	^46^
**30** (NH2)	HDAC6	5	Nexturastat A	CRBN	PEG-triazole-alk (5)	HDAC6–PROTAC–CRBN ternary complex possesses large flexibility	^47^
**31** (12d)	HDAC6	18	Nexturastat A	CRBN	Alk-triazole-alk (18)	The optimal number of methylene units in the linker is about 6, and the C4-linked series are slightly more potent than the C5-series; the triazole ring linked to C4 contributed to the induced interaction between CRBN and IKZFs	^48^
**32** (3j)	HDAC6	11	Nexturastat A	**VHL,** CRBN	Alk-triazole-alk (11)	Degraders with PEG linkers not active; Increased potency with higher number of methylene units; The linker length required for VHL-based PROTACs is much longer than for CRBN-based degraders; VHL-based degrader more potent in HDAC6 degradation and tubulin acetylation than CRBN-based degrader	^49^
**33** (11b) **34** (13f)	HDAC6	9	Vorinostat Nexturastat A	CRBN	Alk (3) Alk-triazole (6)	The potency of degraders increases with increasing linker length; Replacing the amino group with an alkyne or phenyl group directly attached to thalidomide increases potency and selectivity towards HDAC6	^29^
**35** (4)	HDAC1 HDAC6	1	Vorinostat-like	CRBN	Alk-PEG (1)	–	^50^
**36** (A6) **37** (B4)	HDAC6	11	Vorinostat-like (A) Benzeidazole (B)	CRBN	PEG (8) Alk (2) Other (1)	PROTACs containing 8-aminooctanoic acid as linker demonstrated the most potent degradation of HDAC6 and the strongest hyperacetylation of α-tubulin	^32^
**38** (14a)	HDAC6	8	Indirubin derivative	CRBN	Triazole-PEG (8)	**14a** with the shortest 1PEG linker is the most potent; **14e** with the longest 5PEG linker is the second in terms of degrading potency *D*_max_	^51^
**39** (1) **40** (4)	HDAC6	6	Difluoromethyl-1,3,4-oxadiazole	CRBN VHL	PEG (4) Alk (2)	The most potent 1 and 4 degraders are derived from HDAC6-targeting warhead modified in *meta*-position of solvent exposed phenyl ring with linker	^31^
**41** (TH170)	HDAC6	6	TH74 SD100NC	CRBN	Alk (3) Alk-triazole (3)		^52^
**42** (XY-07-035) **43** (XY-07-093) **44** (XY-07-187)	Pan-HDAC	48	Vorinostat Dacinostat TMP269 NVS-HD1	VHL CRBN IAP	Alk (25) PEG (23) –	HDAC degradation is lost with the PEG4 linker, while PEG3 and PEG5 analogs in VHL series were active; potency lost with the longest linker PEG5 in the CRBN set; VHL-recruiting series favoured HDAC3; CRBN-recruiting series favoured HDAC6 and 8 degradation; changing the ligase to cIAP changes the HDAC isoform selectivity to HDAC6 and results in collateral degradation of CoREST	^30^
**45** (4c)	HDAC8	4	NCC-149 analog	CRBN	Alk (4)	C8 or C11 linker attached at the *meta*-position is important for HDAC8-degradation	^53^
**46** (ZQ-23)	HDAC8	37	BRD73954	CRBN	Alk (28) Other (9) (diamine conjugated with omega-hydroxycarboxylic acid)	The flexible two-part linker used, with one of the longest linkers (12 atoms in total) optimal for the HDAC8 degradation	^54^
**47** (CRBN_1e)	HDAC8	12	benzhydroxamates -based	**CRBN** VHL	Alk (6) PEG-alk (3) Alk-triazole (3)	VHL-based PROTACs did not show significant HDAC8 degradation	^55^
**48** (SZUH280)	HDAC8	18	PCI-34051	**CRBN** VHL	Alk (4) Alk-triazole (5) PEG (4) PEG-triazole (5)	Medium-length alkyl linkers induced the highest HDAC8 degradation; replacing the carbon with the oxygen atom caused more pronounced HDAC8 degradation observed with shorter or longer linkers	^56^
**49** (CT-4)	HDAC8	4	4	CRBN	Alk (4)	Shortening the linker reduced the potency by a factor of 10 or more	^57^

^a^The most active in bold.

**Table 5.** Experimental methods and cell models applied in development of KDAC-PROTACs.

PROTAC	Target	Cell lines	Cell proliferation/cell cycle/apoptosis	Target degradation	Other assays	Ref
**13** (12)	SIRT2	HeLa		Western blot	*In vitro*: fluorogenic HDAC activity; immunofluorescence microscopy	^14^
**14** (TM-P4-Thal)	SIRT2	MCF7, MDA-MB-231, MDA-MB-468, BT-549, K-Ras4a-expressing, HEK293T		Western blot; sirtuins degradation in cells; mass spectrometry	*In vitro:* HPLC sirtuin diacylation; K-Ras4a de-fatty acylation in cells; SIRT2 deacetylation activity in cells	^37^
**15** (PRO-SIRT2)	SIRT2	HEK293		Western blot	*In vitro*: HPLC sirtuin deacylation assay	^38^
**16** (4)	HDAC1/2/3	E14, HTC116	Flow cytometry	Western blot	*In vitro:* HDAC inhibition with the CoREST Complex	^39^
**17** (JPS014) **18** (JPS016) **19** (JPS036)	HDAC1/2/3	HCT116	CellTiter-Glo; flow cytometry	Western blot	*In vitro:* RNA-seq	^40^
**20** (JPS026) **21** (JPS004)	HDAC1/2/3	HTC116	Flow cytometry	Western blot	*In vitro:* RNA-seq	^41^
**22** (JMC-137)	HDAC1/2	HCT116		Western blot	*In vitro:* HDAC inhibition with the CoREST Complex	^42^
**23** (2)	HDAC1/2/3	HEK293T	MTT	Western blot	*In vitro:* fluorogenic HDAC activity	^43^
**24** (XZ9002)	HDAC3	MDA-MB-468, T47D, HCC1143, BT549	CellTiter-Glo	Western blot	*In vitro:* HDAC activity (HDAC-Glo I/II); colony formation assay	^44^
**25** HD-TAC7	HDAC3	RAW264.7	MTS	Western blot	In vitro: fluorogenic HDAC activity; RT-qPCR	^28^
**26** (7) **27** (11)	HDAC4	Jurkat E6-1, Neuro 2a, Q175, SH-SY5, MDCK, MDR1, BCRP	Hoechst and DRAQ7 imaging	Western blot; ELISA; Meso Scale Discovery	*In vitro*: fluorogenic HDAC activity (*in vitro* and in cells); kinetic solubility assay	^45^
**28** (9c)	HDAC6	MCF7, MM.1S		Western blot		^15^
**29** (NP8)	HDAC6	HeLa, A549, hTERT-RPE1, U251, Jurkat, HCT116, RPMI 8226, MM.1S	CCK-8	Western blot; fluorescence microscopy		^46^
**30** (NH2)	HDAC6	Hela, MM.1S, Mino, Jeko-1, HUVEC, MDA-MB-231		Western blot		^47^
**31** (12d)	HDAC6	Hela, HepG2, A375, A431, MCF7, MM.1S, RPMI8226, RS4;11, Jurkat	Resazurin	ELISA; Western blot	*In vitro:* RT-qPCR	^48^
**32** (3j)	HDAC6	MM.1S, 4935, HEK293T, U87MG, A549, MCF7		ELISA; Western blot		^49^
**33** (11b) **34** (13f)	HDAC6	MM.1S, Jukart	Resazurin	ELISA; Western blot		^29^
**35** (4)	HDAC1 HDAC6	HL60		Western blot	*In vitro*: fluorogenic HDAC activity	^50^
**36** (A6) **37** (B4)	HDAC6	HL-60, U266, MOLM-13, MV4-11, THP-1, KASUMI-1, 697, REH, K562, HDAC6-fused, HiBit K562	CellTiter Glo 2.0; Caspase 3/7; Annexin V/PI; flow cytometry	Western blot; mass spectrometry	*In vitro:* fluorogenic HDAC activity	^32^
**38** (14a)	HDAC6	K652, HeLa, TPH-1	CCK-8	ELISA; Western blot	*In vitro:* fluorogenic HDAC and CDK activity; inflammasome activation *In vivo:* LPS challenged C57BL/6 mice (serum levels of IL-1β and TNF-α; H&E stained lung tissues)	^51^
**39** (1) **40** (4)	HDAC6	MM.1S	CellTiter Glo 2.0	Western blot	*In vitro:* fluorogenic HDAC activity	^31^
**41** (TH170)	HDAC6	SK-N-BE(2)-C		Western blot	*In vitro*: fluorogenic HDAC activity assays	^52^
**42** (XY-07-035) **43** (XY-07-093) **44** (XY-07-187)	Pan-HDAC	KELLY, MM.1S, HEK293T	CellTiter Glo	Western blot; HDAC8 cellular degradation; mass spectrometry	*In vitro*: fluorogenic HDAC activity; competitive displacement assay for cellular CRBN and VHL engagement	^30^
**45** (4c)	HDAC8	Jurkat	Alamar Blue		*In vitro*: fluorogenic HDAC activity	^53^
**46** (ZQ-23)	HDAC8	HT116		Western blot		^54^
**47** (CRBN_1e)	HDAC8	SK-N-BE(2)-C, IMR-32, HEK293	Trypan blue; Alamar Blue	Western blot	*In vitro:* colony formation assay; cell differentiation assay	^55^
**48** (SZUH280)	HDAC8	A549, HeLa, HCT116, MDA-MB-231, HBEC3-KT, Jurkat	CCK-8; Annexin V FITC; flow cytometry	Western blot; ELISA; mass spectrometry	Clonogenic Assay; immunofluorescence staining; RT-qPCR; RNA-Seq *In Vivo:* NOD/SCID mice (tumour xenograft studies with A549 cells) (H&E staining, immunohistrochemistry)	^56^
**49** (CT-4)	HDAC8	MDA-MB-231, Jurkat	MTS; Caspase 3/7, Annexin V-FITC	Western blot	*In vitro*: fluorogenic HDAC activity; migration scratch assay	^57^

SIRT2 is a prospective target for pharmaceutical intervention since it has been linked to the pathogenesis of bacterial infections, neurological disorders, and many cancers. Novel CRBN recruiting PROTACs that may effectively degrade SIRT2 in a variety of breast cancer cells have been published recently. Hong et al. utilized a selective SIRT2 inhibitor containing thiomyristoyl lysine that generates a covalent intermediate with NAD^+^. TM-P4-Thal (**14**) specifically degraded SIRT2 in MCF7, BT549, MDA-MB-231, and MDA-MB-468 breast cancer cell lines. In addition to its deacetylation function, SIRT2 is effective at removing long-chain fatty acyl groups from lysine, e.g. defatty-acylating K-Ras4a to increase K-Ras-mediated transformation. Both SIRT2 deacetylase and defatty-acylase activities were inhibited by **14** in living cells. This phenomenon may be responsible for the stronger cytotoxicity of **14** as compared with SIRT2 inhibitor illustrating a further benefit of the PROTAC over the conventional inhibition strategy^37^.

**Figure 4.**
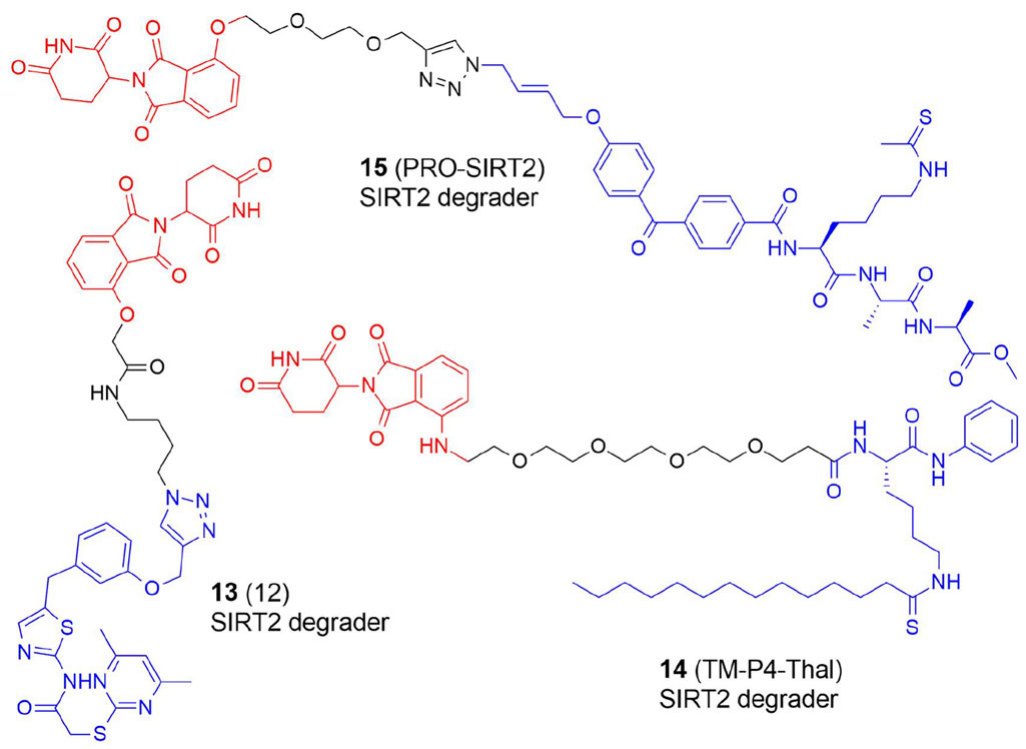
Structures and activities of PROTACs targeting SIRT2.

Azido-containing activity-based chemical probes (ABPs) elaborated to PROTACs *via* “click” conjugation to thalidomide have been reported recently^38^. ABPs contained a simple Ala-Ala-Lys tripeptide backbone with a thioacyl “warhead”, a photoaffinity group (benzophenone or diazirine), and a bioorthogonal group (terminal alkyne or azido) enabling reporter conjugation. The synthesized PRO-SIRT2 PROTAC (**15**) significantly lowered the endogenous level of SIRT2 in HEK293 cells (Figure 4).

### PROTACs targeting lysine deacetylases: HDAC1/2/3

**Figure 5.**
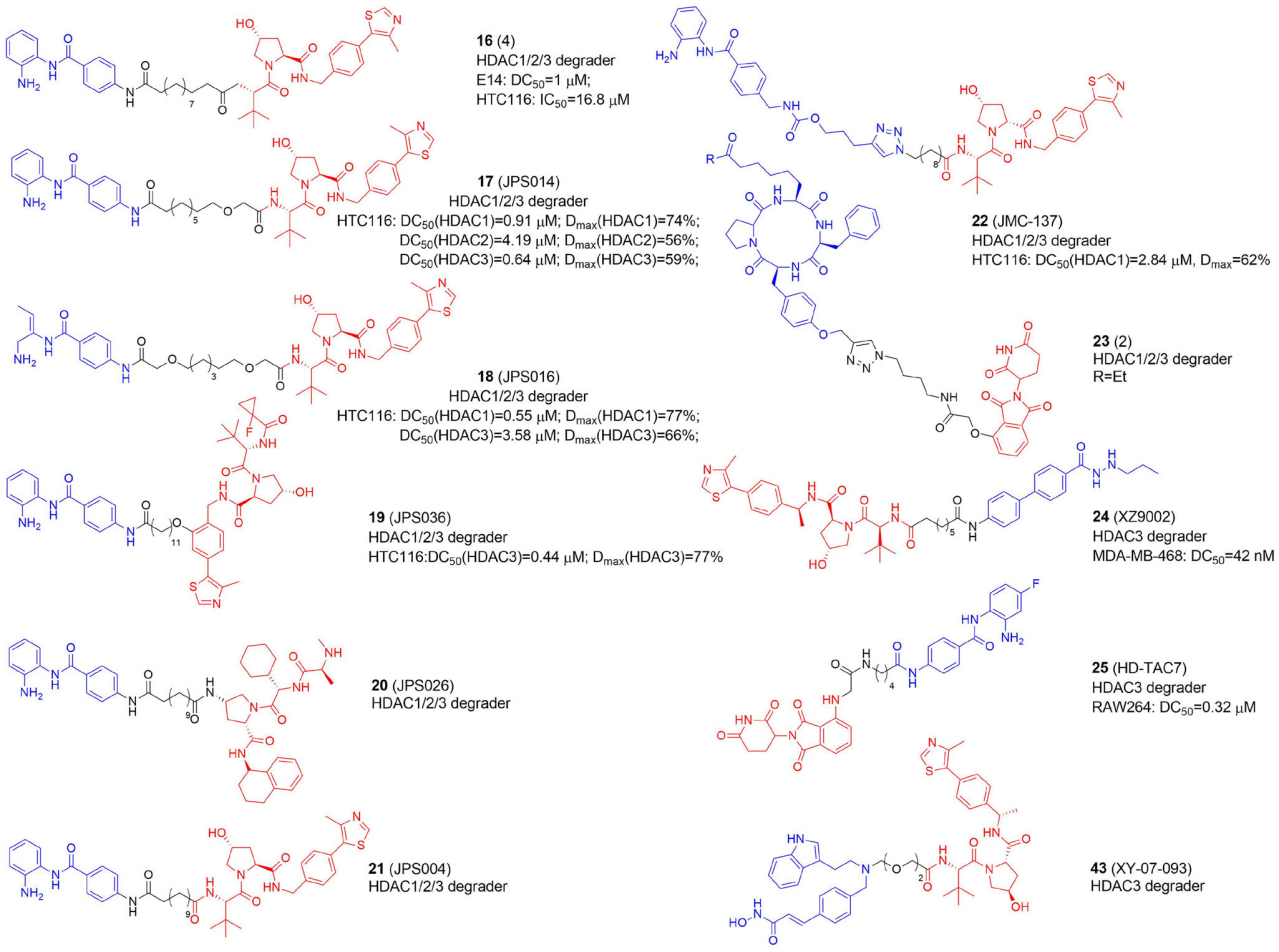
Structures and activities of PROTACs targeting HDAC1, HDAC2, and HDAC3.

Class I HDACs have also been the focus of various investigations. In 2020 first PROTACs targeting HDAC1/2/3^39^^,^^43^ or displaying preference against HDAC3^28^^,^^44^ were reported (Table 4 and 5). Smalley et al. synthesized CRBN- and VHL-based PROTACs with CI-994, a benzamide-based HDAC1/2/3 inhibitor^39^. PROTACs bearing longer linkers with at least 12 atoms were cell permeable more effective HDAC1/2/3 degraders than compounds with shorter linkers. The most active degrader 4 (**16**) (Figure 5) contained VHL E3 ligand. Further optimization studies within this research group discovered novel PROTACs with differing selectivity profiles between HDAC 1, 2, and 3^40^. By modifying CI-994, linker, and ligand, the authors identified submicromolar PROTACs JPS014 (**17**), JPS016 (**18**), and JPS036 (**19**) for HDAC1 and/or HDAC3 in HCT116 cells. Interestingly, due to the incorporation of oxygen atoms into the alkyl chain, HDAC1 and HDAC3 selectivity was achieved, while the addition of a fluoro-cyclopropyl group to the VHL ligand targeted HDAC3. HDAC1/2 degradation by PROTACs correlated with increased global gene expression and apoptosis. Subsequently, the authors defined a novel class of HDAC1/2/3 degraders (JPS026, **20**) by coupling CI-994 to the IAP ligand via a 12-carbon linker. **20** greatly increased the sensitivity of HCT116 cells to apoptosis as compared to VHL-derived PROTACs or CI-994 inhibitor. PROTACs with VHL ligands were the most potent to promote histone hyperacetylation. The level of histone acetylation was greater with longer linkers (JPS004 (**21**) > JPS026). RNA sequencing of PROTACs-treated HCT116 cells revealed a distinctive gene expression signature in which the DNA replication machinery and cell cycle are suppressed. The number of differentially expressed genes and the more effective HDAC modulators (those best capable to cause histone hyperacetylation) were correlated^41^.

The Hodgkinson group also synthesized class I HDAC PROTACs based on the selective HDAC1/2/3 inhibitor entinostat (MS-275) currently undergoing clinical trials for the treatment of solid tumours and hematological cancers. They prepared entinostat analogs with a fluorine atom on the benzamide ring, which is structurally similar to tucidinostat. With a “click” chemistry approach the authors produced class I HDAC1/2/3 degraders (JMC-137, **22**) but potency appeared to be limited probably due to low cell permeability. Also, using shorter alkyl linker (<12 atoms) together with the carbamate and triazole groups, as closer analogs of entinostat, did not improve PROTACs’ potency^42^.

Instead of CI-994, Roatsch et al. in turn employed “click” chemistry to introduce macrocyclic tetrapeptide HDACi into CRBN-recruiting HDAC1/2/3 degraders^43^. Macrocyclic peptide inhibitors, which include apicidin, trapoxin, and the family of azumamides are typically thought to be less effective zinc binders than hydroxamic acid or o-aminobenzamide. A trapoxin scaffold was used to synthesize ethyl ketone-containing PROTACs 1 and 2 and hydroxamate-containing compounds 3 and 4. Compound 2 (**23**) appeared to be the most potent HDAC degrader without overall cytotoxicity observed in HEK293T cells.

HDAC3-selective VHL- and CRBN-recruiting PROTACs were developed by Xiao et al. using benzoylhydrazide SR-3558^44^. The most active VHL-based degrader (8c: XZ9002, **24**) induced HDAC3 degradation in MDA-MB-468 cells. Independently to Smalley et al., Cao et al. also utilized CI-994, however, HDAC3 selectivity was achieved by using a para-fluoro ortho-amino anilide core^28^. The most potent and selective HD-TAC7 (**25**), in contrast to the HDACi, did not significantly influence the gene transcription of IL-6, IL-10, iNOS, and TNFα in RAW 264.7 macrophages.

### PROTACs targeting lysine deacetylases: HDAC4

**Figure 6.**
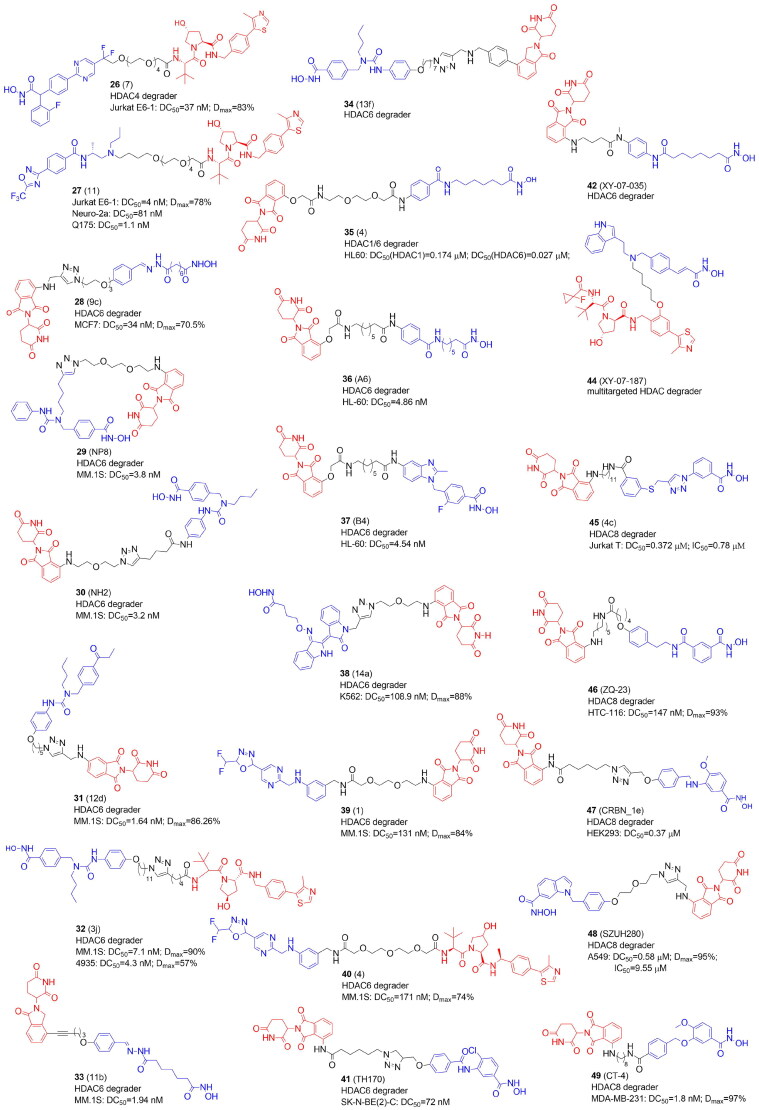
Structures and activities of PROTACs targeting HDAC4, HDAC6, and HDAC8.

Besides cancer, the inhibition of HDACs has been proposed as a promising strategy to treat neurodegenerative disease. Hydroxamic acid-based PROTAC 7 (**26**) (Figure 6) and the trifluoromethyloxadiazole-derived compound 11 (**27**) were the first reported VHL-based degraders that selectively and potently degraded HDAC4 in primary neurons at low nanomolar range^45^. **26** and **27** demonstrated HDAC4 preference over HDAC5, HDAC7, and HDAC9.

### PROTACs targeting lysine deacetylases: HDAC6

HDAC6, the largest among the HDACs, promotes the development of tumours and oncogenic transformation by enabling proliferation as well as cancer cell migration and invasion. As possible therapies for the treatment of malignancies and neurological illnesses, HDAC6-selective inhibitors have been created. These HDAC6-selective inhibitors can inhibit the C-terminal catalytic domain. However, HDAC6 also has an N-terminal catalytic domain and a zinc-finger ubiquitin binding domain^22^. In 2018 the first degrader for zinc-dependent HDACs was designed and selective degradation of HDA6 was achieved (over HDACs 1, 2, and 4) using non-selective hydroxamate-based pan-HDAC inhibitor AB3, conjugated with the ligand of CRBN E3 ligase^15^. The most potent PROTAC 9c (**28**) evoked the maximal effect of HDAC6 degradation at as low as 80 nM in the MM.1S cell line and 123–370 nM in MCF7 cells. In further studies, **28** appeared to be more potent in transformed patient-derived ovarian cancer than ricolinostat (ACY-1215), an HDAC6 inhibitor in phase I and II clinical trials^58^. These results raise the intriguing question of whether it is better to create highly effective and selective HDAC6 degraders using selective or unselective HDAC6 inhibitors. However, HDAC6 degraders developed by Yang et al.^15^ have several limitations. The hydrazone linker, for example, is not hydrolytically stable and so unsuitable for further research. Although **28** selectively degraded HDAC6, the pan-inhibitor warhead still inhibited other HDACs, as evidenced by the increased level of acetylated histones. Therefore, other HDAC6 degraders have been released in the next year based on Nexturastat A (Next-A), an HDAC6-selective inhibitor that binds to the catalytic pocket through a Y-shaped conformation^46–48^.

To create novel HDAC6-targeting PROTACs, An et al. inserted pomalidomide onto the end of the aliphatic chain of Nex-A via various PEG linkers. NP8 (**29**) was the most efficient degrader triggering HDAC6 degradation at 100 nM in many cell lines, most notably in multiple myeloma MM.1S cells. However, PROTACs had no better antiproliferation effect than Next-A^46^.

In the same year, the Rao group reported other HDAC6-degrading PROTACs with pomalidomide attached at the benzene ring of Next-A through a triazole carbon linker. When compared with **29**, the most potent NH2 (**30**) exhibited comparable degradation activity in MM.1S cells despite **28** and **29** being extended from different directions of Next-A, which confirms the large flexibility of the ternary complex^47^.

By attaching Next-A to pomalidomide, the Tang group synthesized 18 degraders divided into two groups based on the amino group’s connecting position on the phthalimide ring of pomalidomide: C4- or C5-linked series. Degrader 12d (**31**) belonging to the C4-series demonstrated the highest potency for the degradation of HDAC6 (DC_50_=1.6 nM in MM.1S cells). When compared to the previously developed **28, 31** demonstrated significantly improved selectivity for increasing the level of acetylated tubulin over acetylated histone H3, indicating the benefit of replacing a pan-HDAC inhibitor with an HDAC6 selective inhibitor as the HDAC6 ligand for PROTACs. Besides, the advantage of **31** over Next-A, is the synergistic effects of HDAC6 degradation/inhibition strengthened by degradation of Ikaros family zinc finger proteins 1 and 3 (IKZF1/3). Pomalidomide analogs are known to activate CRBN's E3 ligase activity towards the IKZFs and promote their ubiquitination and subsequent degradation and due to this activity are thought to have a significant antiproliferation effect on multiple myeloma. Preserving IKZFs’ degradation activity by **31** would have improved antimyeloma activity^48^.

Subsequently, based on earlier research, the Tang group developed a new class of selective HDAC6 degraders using Next-A and substituting VHL instead of CRBN. Due to the distinct E3 ligase that was recruited, and therefore, formation of the different ternary complex, a substantially longer linker was necessary than for PROTACs that attracted CRBN. The most potent compound 3j (**32**) showed nanomolar degradative activity and broad generality for the degradation of HDAC6 in various human and mouse cell lines, including MM.1S, HEK293T, U87MG, A549, MCF7, and 4935 cells^49^. Meanwhile, they created a high throughput in-cell ELISA assay to evaluate the binding affinity of a library of thalidomide analogs to E3 ligase. The novel derivatives were used to design two series of CRBN-recruiting PROTACs. Activities of compounds 11b (**33**) with a short, 5-atom long rigid linker and 13f (**34**) bearing the phenyl substituent on the phthalimide, and longer linkers were comparable with the previous PROTAC from this group with a triazole ring and more than six methylene units^48^ in terms of the degradation of HDAC6^29^.

Sinatra et al. developed an approach using hydroxamic acids immobilized on resins (HAIRs) to synthesize the library HDACi. Compound 4 (**35**) degraded HDAC6 and HDAC1 in a concentration-dependent manner in a leukemic HL60 cell line^50^. The same group developed two series of PROTACs to test whether a selective or unselective HDACi is advantageous for selective HDAC6 degradation and effective antileukemic activity^32^. They used an unselective vorinostat-like HDACi based on an alkyl linker or a selective benzimidazole-based HDAC6 inhibitor^59^ using a combination of a solution- and solid-phase methodology. From each series potent and selective HDAC6 degraders (A6 (**36**) and B4 (**37**), respectively) were developed, showing in leukaemia cell lines DC_50_ values of 3.5 and 19.4 nM and comparable *D*_max_ values over 80%.

In 2021, the He group conjugated a CDK/HDAC6 inhibitor derived from a natural product, indirubin, with pomalidomide to create HDAC6 degraders blocking NLRP3 inflammasome activation. The most potent compound 14a (**38**) bearing the shortest linker decreased the level of HDAC6 without any obvious degradation of HDAC1 and CDK2 in K562, HeLa, and activated THP-1 cells. Importantly, the CDK/HDAC6 inhibitor itself was more cytotoxic than **38**. Intraperitoneal injection of **38** in C57BL/6 mice blocked NLRP3 inflammasome activation confirming that NLRP3 inflammasome activation depends on HDAC6 in vivo^51^.

The first non-hydroxamate selective HDAC6 degraders containing difluoromethyl-1,3,4-oxadiazole warheads as ZBGs were reported by Keuler et al.^31^ Compound 1 (**39**) from the CRBN-recruiting series caused the most sufficient degradation whereas compound 4 (**40**) showed the greatest decrease in HDAC6 levels among the VHL-recruiting PROTACs in MM.1S cells.

The latest study describing the synthesis of HDAC6 targeting PROTACs comes from the Sippl group^52^. They designed PROTACs based on the previously synthesized HDAC6 selective inhibitors, benzohydroxamates TH74 and SD100NC. The most active pomalidomide-based PROTAC TH170 (**41**) evoked strong HDAC6 degradation in the SK-N-BE(2)-C neuroblastoma cell line.

A global quantitative chemo-proteomics study mapped the PROTAC-mediated degradation of zinc-dependent HDAC isoforms with a library of 48 pan-HDAC PROTACs^30^. Vorinostat and dacinostat were selected for targeting class I and IIb HDACs while TMP269 and NVS-HD1 were for class IIa. Also, three different ligands were selected to recruit different E3 ligases (CRBN, VHL, and IAP). HDAC6 was most commonly degraded, followed by HDAC8 and HDAC3. Interestingly, HDAC3, 6, and 8 were characterized by the longest half-lives. HDAC9 was not degraded at all, while HDAC1, 4, and 2 were degraded to a small extent. Also, the library of dacinostat-based PROTACs revealed that the VHL-based PROTACs strongly preferred HDAC3, but the CRBN recruiting PROTAC preferred HDAC6 and 8. IAP-recruiting compound specifically degraded HDAC6, however with weak potency. XY-07–035 (**42**) showed significant degradation of HDAC6, XY-07–093 (**43**) - HDAC3, and XY-07–187 (**44**) was multi-targeting (HDAC3-, 6-, and 8). Importantly, collateral degradation of other components within HDAC protein complexes was also observed, highlighting the concerns of indirect targets in the development of HDACs targeting PROTAc.

Also worth mentioning are the studies from the Zhang group targeting HDAC6 degradation to highlight how important it is to plan appropriate control experiments in PROTAC research^60^. Three hydroxamic acids containing the bestatin amide scaffold as cIAP1 ligands were synthesized, however the observed inhibitory and decreasing activity against HDACs 1, 6, and 8 was not due to protein degradation.

### PROTACs targeting lysine deacetylases: HDAC8

The first HDAC8-selective PROTACs have been disclosed in 2022^53^. The discovery of histone and non-histone HDAC8 substrates like p53, the structural maintenance of chromosomes 3 protein (SMC3), estrogen-related receptor α (ERRa), and AT-rich interactive domain-containing protein 1 A (ARID1A) suggests that this enzyme has a complicated role in maintaining cellular homeostasis. It’s therefore not surprising that overexpression of HDAC8 was found to be associated with cancer development. Chotitumnavee et al. employed an analog of HDAC8-selective inhibitor NCC-149 and CRBN ligand to synthesise PROTACs. Compound 4c (**45**) with a C11 linker exhibited the most potent and selective HDAC8-degrading activity and inhibited the growth of T-cell leukaemia Jurkat cells.

Very soon the Chen group developed pomalidomide-based HDAC8-targeting PROTACs using dual HDAC6/8 inhibitor BRD73954^54^. Compound ZQ-23 (**46**) (Figure 6) exhibited the most potent HDAC8 degrading activity in HCT-116 cells.

Darwish et al. designed PROTACs based on benzhydroxamates, with low nanomolar range inhibitory activity against HDAC8, combined with either CRBN or VHL ligands^55^. The selectivity of benzhydroxamates can be related to the fact that the aromatic capping group occupies an HDAC8-specific pocket, which is lacking in the other HDAC isoforms. Also, PEG-, alkyl-based, and triazole ring-containing linkers were used. Nearly all compounds demonstrated HDAC8 inhibitory activity and weak cytotoxicity against HEK293 cells. CRBN-based HDAC8 PROTACs with a triazole linker CRBN_1e (**47**) showed the strongest effect on colony formation neuroblastoma SK-N-BE(2)-C cells with MYCN amplification and non-functional p53. **47** also showed good potency in HDAC8 degradation and a strong hyperacetylation of the HDAC8 substrate SMC3. Finally, a combination of **47** with neuronal differentiation inducer retinoic acid promoted the differentiation phenotype of SK-N-BE(2)-C cells.

Zhou’s group reported the antitumor activity of HDAC8 PROTACs in solid tumours^56^. They linked an indole-based HDAC8-selective inhibitor PCI-34051 with CRBN or VHL ligands and selected CRBN-based compound 16e (SZUH280) (**48**) with a short PEG linker as the effective HDAC8 degrader in the 549 human lung cancer cell line. In an A549 tumour mouse model, **48** induced HDAC8 degradation and tumour regression, especially, in combination with irradiation.

Dekker’s group reported a class of HDAC8 PROTACs by connecting compound 4 and pomalidomide with flexible aliphatic linkers of various lengths^57^. Among them, CT-4 (**49**) was identified as a potent HDAC8 degrader with single-digit nanomolar DC_50_ values in both MDA-MB-231 cells and Jurkat cells. **49** demonstrated moderate selectivity (the 20-fold difference in DC_50_) between HDAC8 and HDAC6, but good selectivity over HDAC1 and HDAC3. In MDA-MB-231 cells, **49** showed strong anti-migration activity but only weak anti-proliferative activity. In contrast, in Jurkat cells, **49** successfully caused apoptotic cell death.

Firstly, VHL recruiting PROTACs were found to be more effective E3 ligands in degrading class I HDACs while CRBN ligands had a preference towards HDAC 6 and 8. IAP-based degraders exhibited rather weak degradation efficiency. Secondly, the HDACi motif of an HDAC-PROTAC is critical for degradation selectivity. Most HDACi have a zinc-binding group-linker-cap group pharmacophore, where ZBG coordinates to the conserved Zn^2+^ ion found in the active sites of all HDACs. While the cap group binds to the surface of the HDAC, the extended linker often imitates the protruding acetylated lysine side chain of a histone substrate. Interestingly, unselective HDACi inhibitors incorporated into PROTACs tend to show degradation preference for the particular HDAC class. Thirdly, the linker is also critical for degradation efficacy and selectivity. Most of the linkers in HDAC PROTACs are flexible alkyl chains or PEG. However, the design of a universally applicable linker is not possible as the specific HDAC class of interest, the HDCi, and recruited E3 affect the final effect together.

## Methylation

### Protein lysine methyltransferases and arginine methyltransferases

Protein lysine methyltransferases (PKMTs, KMTs) and protein arginine methyltransferases (PRMTs) catalyze the covalent linking of a methyl group to the ϵ‐amino group of lysine and guanidine group of the arginine residue. Unlike KMTs and PRMTs, which methylate side chains, protein N-terminal methyltransferases (NTMTs) transfer methyl groups to protein N-terminal α-amino groups. KMTs may be divided structurally into subfamilies which often share common SET (Su(var)3–9, enhancer of zeste and trithorax) and the seven-beta-strand (7βS) domain (Table 6)^61^. Protein lysine methylation, like other PTMs, is reversible and can be reversed by a different class of enzymes called protein lysine demethylases (KDMs; also known as erasers).

**Table 6.** Categorisation of human KMTs, PRMTs, and NTMTs.

Lysine methyltransferases				Arginine methyltransferases		N-terminal methyltransferases	
Family	Subfamily	Protein name	Aliases	Type	Protein name	Protein name	Aliases
DOT1-lik	DOT1L			I	PRMT1	**NTMT1**	METTL11A, NRMT
SET domain	SET1 family	MLL1	KMT2A		PRMT2	NTMT1	METTL11B, NRMT2
		MLL2	KMT2B		PRMT3	METTL13	eEF1A-KNMT
		MLL3	KMT2C		PRMT4		
		MLL4	KMT2D		PRMT6		
		SET1A	KMT2F		PRMT8		
		SET1B	KMT2G	II	**PRMT5**		
	SET2 family	NSD1	KMT3B		PRMT9		
		**NSD2** ^a^	KMT3G	III	PRMT7		
		**NSD3**	KMT3F				
		SETD2	HYPB, HIF-1				
		ASH1L	KMT2H				
	EZH family	EZH1	KMT6B				
		**EZH2**	KMT6				
	SUV39 family	SUV39H1	KMT1A				
		SUV39H2	KMT1B				
		G9A	KMT1C				
		GLP	KMT1D				
		SETDB1	KMT1E, ESET				
		SETDB2	CLLL8, KMT1F				
	RIZ family	RIZ1	KMT8, PRDM2				
		BLIMP1	PRDM1				
		PFM1	CRS2				
	SMYD family	SMYD1	KMT3D				
		SMYD2	KMT3C				
		SMYD3	KMT3E				
		SMYD4					
		SMYD5					
	Others	SET8 SUV4-20H1	KMT5A KMT5B				
		SUV4-20H2	KMT5C				
		SET7/9	KMT7				

^a^Enzymes for which PROTACs have been developed are marked in bold

**Figure 7.**
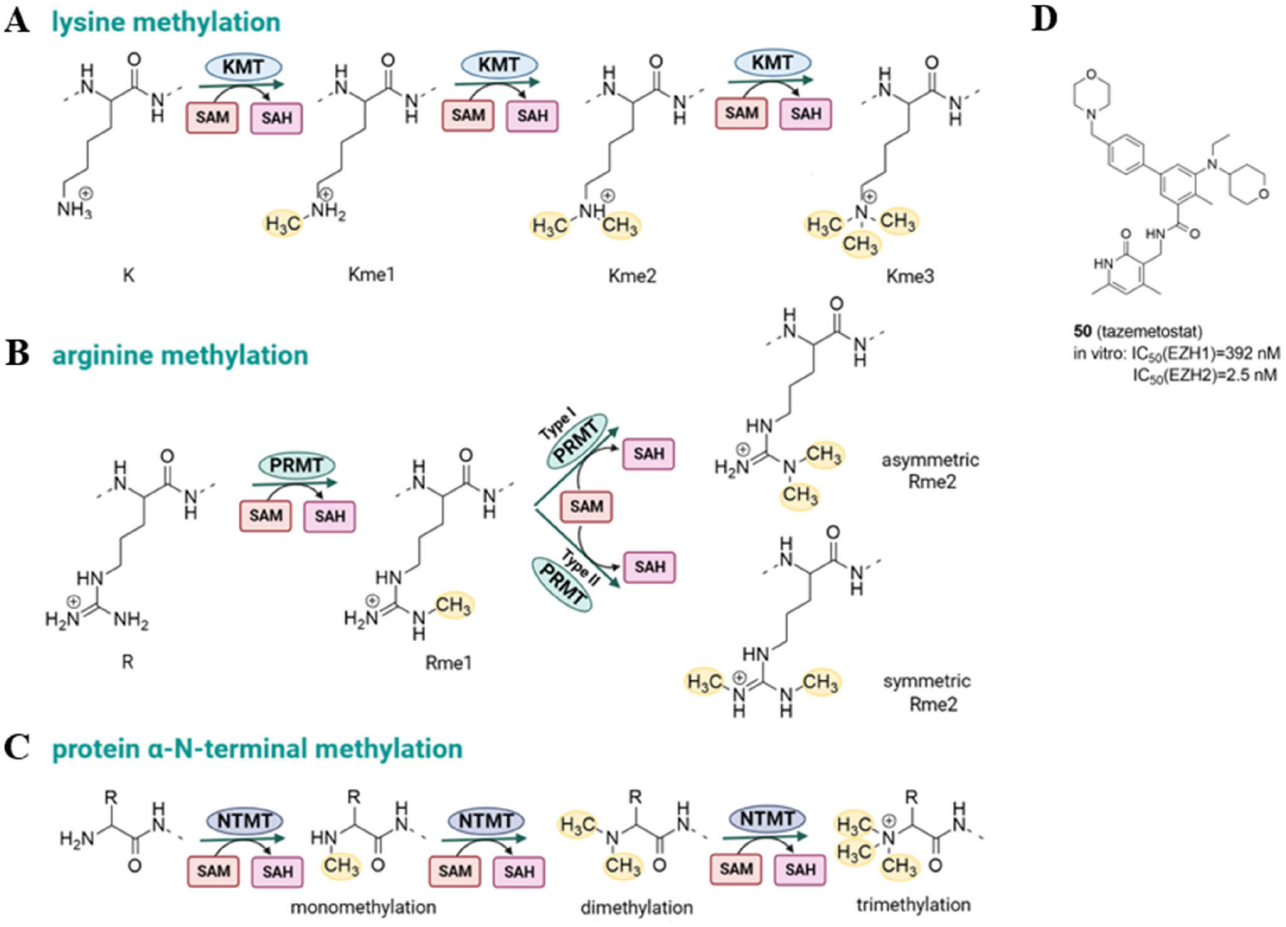
Protein lysine methylation catalyzed by lysine methyltransferases (KMTs) (A); protein arginine methylation catalyzed by protein arginine methyltransferases (PRMTs) (B); N-terminus methylation catalyzed by N-terminal methyltransferases (NTMTs) with S-adenosyl methionine (SAM) as the methyl group donor (C); tazemetostat – KMT inhibitor approved by FDA (D).

Protein lysine methylation is the chemical modification, which occurs by the addition of one, two, or three methyl groups (Kme1, Kme2, or Kme3, respectively) from the S-adenosyl-l-methionine (SAM) cofactor (Figure 7(A)). In contrast to acetylation, lysine methylation has minimal effects on the charge characteristics of the lysine side chain. Instead, lysine methylation largely serves as a signal for the selective recruitment of effector proteins. The canonical lysine methylation sites in humans are located at lysine 4 (H3K4), lysine 9 (H3K9), lysine 27 (H3K27), lysine 36 (H3K36), and lysine 79 (H3K79), as well as lysine 20 on histone H4 (H4K20). The only enzyme that produces H3K79me, one of the few histone modifications present in the globular area of the nucleosome, is the 7βS DOT1L. On the other hand, several enzymes such as EZH1 and EZH2 belonging to Polycomb Repressive Complex 2 (PRC2), G9a and the related G9a-like protein (GLP), SUV39H1, SUV39H2, or SETD7 are involved in the methylation of H3K4, H3K9, and H3K36. Methylation of histone lysines alter chromatin compaction and control gene expression, genomic stability, and the development of cell lineages^62^.

Protein arginine methylation is catalyzed by a family of nine arginine methyltransferases (PRMT1-PRMT9) to generate monomethyl-arginine, asymmetric dimethyl-arginine, and symmetric dimethyl-arginine (Figure 7(B)). Type I PRMTs catalyze the synthesis of monomethyl-arginine (MMA) and asymmetric dimethyl-arginine (aDMA), type II catalyzes the formation of MMA and symmetric dimethyl-arginine (sDMAs), and type III catalyzes only formation of MMA. The fundamental structure of PRMTs is identical with a C-terminal-barrel domain and an N-terminal Rossmann fold where SAM binding takes place. There is no specific arginine demethylase, in contrast to protein lysine demethylases (KDMs)^63^.

The N-terminus can be methylated by N-terminal methyltransferases (Figure 7C). N-terminal methylation is poorly characterized, and to date, only three human N-terminal methyltransferases have been identified. NTMT 1 and NMT2 recognize the X-Pro-Lys consensus sequence (X: any amino acid). The human methyltransferase-like protein 13 (METTL13) methylates both, N‐terminal Gly1 and Lys55 residues in eukaryotic elongation factor 1 alpha (eEF1A)^64^.

Due to the recognized relationship between the dynamics of lysine methylation, gene expression control, and oncogenic programming, small-molecule inhibitors of several KMTs have been the subject of intensive research for both therapeutic and tool applications. Tazemetostat (EPZ6438, **50,** Figure 7(D)) is a first-in-class and the only one so far KMT-targeted epigenetic regulator with the FDA approval for patients with metastatic or locally advanced epithelioid sarcoma and follicular lymphoma that inhibits enhancer of zeste homolog 2 (EZH2) with high potency^65^^,^^66^. However, the upregulation of insulin-like growth factor1 receptor (IGF-1R), mitogen-activated protein kinase kinase (MEK), or phosphoinositide 3-kinase (PI3K) pathways was observed in diffuse large B-cell lymphomas after EZH2 inhibition. What is more, it has been demonstrated that the EZH2 inhibitors GSK126 and tazemetostat were ineffective due to acquired resistance mutations (such as C663Y and Y726F)^67^.

### PROTACs targeting lysine methyltransferases: EZH2

PRC2 is a multicomponent transcriptional repressive complex that catalyzes H3K27me3 methylation. The catalytic EZH1 or EZH2 and three regulatory subunits, embryonic ectoderm development (EED), suppressor of the zeste 12 protein homolog (SUZ12), and retinoblastoma-binding proteins 4/7 (RBBP4/7) make up the core complex of PRC2. Although several small compounds that compete with the EZH2 SAM binding site have entered clinical trials, they are mostly effective against a few cancer types because they are unable to completely stop the oncogenic activity of the PRC2 complex. EZH2's oncogenic function is not solely dependent on its enzymatic activity. Independent of its H3K27 trimethylation activity, tumour proliferation is also correlated with the entire EZH2 protein^68^.

**Figure 8.**
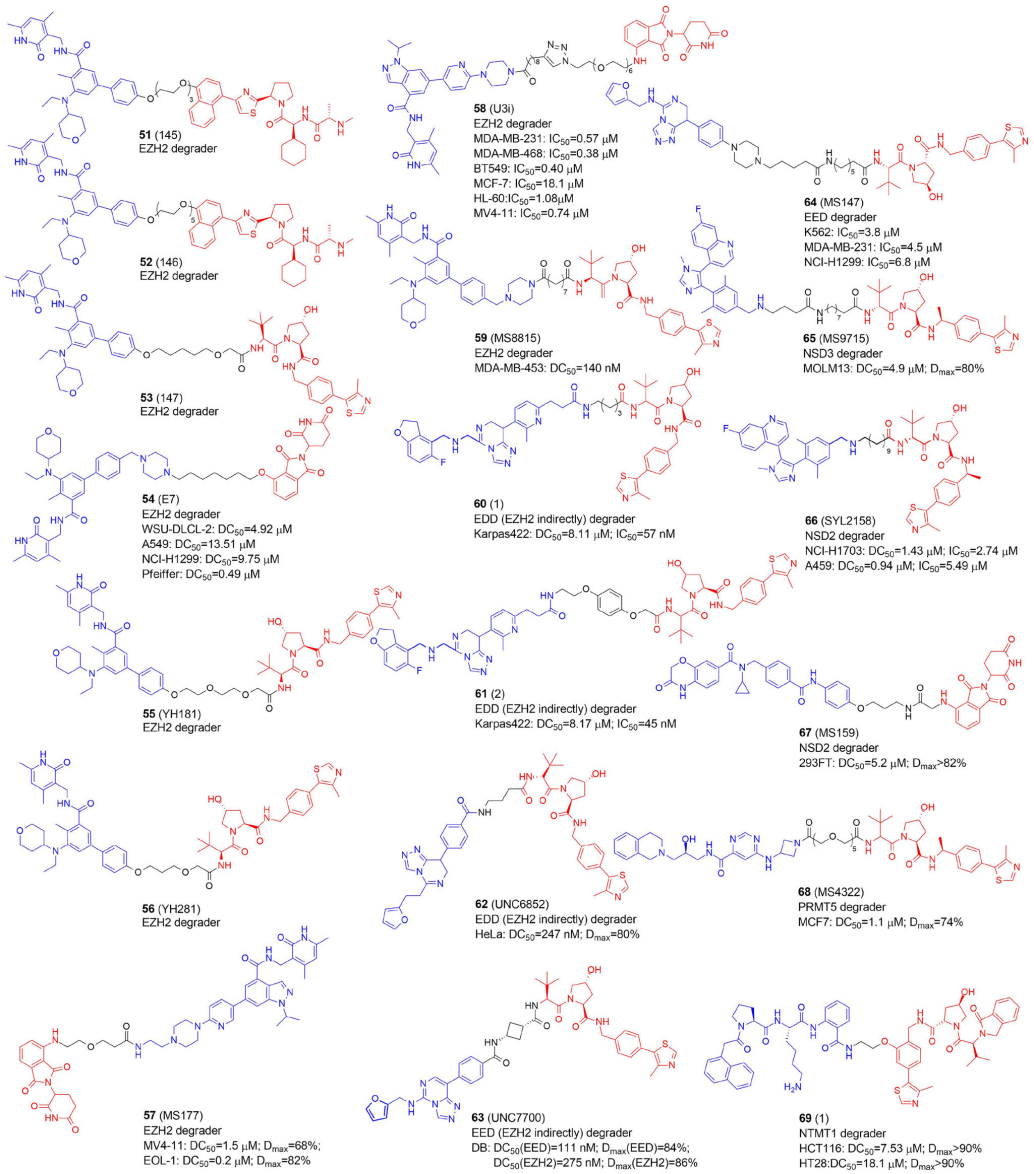
Structures and activities of PROTACs targeting protein methyltransferases.

Recently, a number of PROTACs that either target the EZH2 or EED subunits of the PRC2 complex have been designed (Figure 8, Tables 7 and 8). In 2017, Arvinas patented four moderately effective PROTACs with tazemetostat as a starting point. Compounds **51** and **52** degraded 30%–60% of EZH2 in MDA-MB-231 cells^69^. A year later the same group patented EZH2 degraders containing VHL, CRBN, IAP, and MDM2 ligands. According to the limited biological data available, the most potent degraders were PROTACs with tazemetostat linked at the *para* position of the biphenyl ring through several (di)alkoxy and PEG-like linkers to both VHL and CRBN binders. An exemplary compound **53** led to more than 60% of EZH2 degradation^70^.

**Table 7.** Components and structural details of PROTACs targeting protein methyltransferases.

PROTAC	Target	# of analogs	Original inhibitor	E3 ligase ligands^a^	Type of linker (# of analogs)	Comments	Ref
**51** (145) **52** (146)	EZH2	4	EPZ6438	IAP	PEG (4)	–	^69^ ^,^ ^70^
**53** **(147)**	EZH2	Not specified	EPZ6438	VHL	alkoxyacetamide	–	^70^
**54** (E7)	EZH2	18	GSK126 EPZ6438	CRBN	Alk (18)	The GSK126-based PROTACs with a linker 8-12C demonstrated significant degradation of all PRC2 subunits. Among EPZ6438-based PROTACs, E7 (7C long linker) demonstrated the best degradation efficacies against all PRC2 subunits and a maximum decrease in H3K27me3 levels	^18^
**55** (YM181) **56** (YM281)	EZH2	13	EPZ6438	**VHL** CRBN	PEG-Alk (10) Alk (3)	Only VHL-targeting PROTACs degraded EZH2 with a linker of 7 or 9 atoms length. Longer linkers (14 or 17 atoms) might sacrifice the binding capacity to EZH2, the cell permeability, and ternary complex formation	^71^
**57** (MS177)	EZH2	3	C24	CRBN	PEG (3)	–	^72^
**58** (U3i)	EZH2	28	UNC1999, GSK126, EPZ6438	VHL **CRBN**	Alk (3) Alk-triazole-Alk (7) Alk-triazole-PEG (18)	With the lengthening of carbon chain, the growth inhibitory activity towards TNBC was improved	^73^
**59** (MS8815)	EZH2	16	EPZ6438	VHL	Alk (7) PEG (9)	Compounds with longer linker (6–7 methylene units) were the most active EZH2 degraders	^74^
**60** (1) **61** (2)	EED (EZH2 indirectly)	4	MAK683	VHL	Alk (4)	–	^75^
**62** (UNC6852)	EED (EZH2 indirectly)	6	EED226	VHL	Alk (3) PEG (3)	3-methylene linker optimal; 4-methylene and PEG linkers significant degradation was not seen	^76^
**63** (UNC7700)	EED (EZH2 indirectly)	12	EED226	VHL	Alk (12)	**59** with *cis*-cyclobutane linker was more potent for EZH2 and EED degradation than *trans* isomer	^77^
**64 (**MS147)	EED	10	EED226	VHL	Alk (10)	The difference in degradation profile of MS147, the BMI1 and RING1B degrader, and PROTAC 2 (Hsu et al.), the EED/PRC2 degrader, is most likely due to the linker difference since they share a similar VHL ligand and EED binder	^78^
**65** (MS9715)	NSD3 (MYC indirectly?)	8	BI-9321	**VHL** CRBN	Alk (6) PEG (2)	VHL ligand plus PEG, and short or long alkyl linkers, promoted NSD3 degradation; analogs with CRBN ligand failed to degrade NSD3;	^79^
**66** (SYL2158)	NSD3	26	BI-9321	**VHL** CRBN	Alk (16) PEG (4) Alk-piperazine (6)	Optimal 10 carbon-long alkyl linker; PEG linkers failed to degrade NSD3	^80^
**67** (MS159)	NSD2	13	UNC6934	VHL **CRBN**	Alk (8) PEG (5)	The most active MS159 possesses a (pyridinyl)oxy-propylamine group attached to pomalidomide via a very short alkyl linker	^81^
**68** (MS4322)	PRMT5	8	EPZ015666	VHL	Alk (1) PEG (7)	Compound with a longer PEG linker reduced PRMT5 level	^17^
**69** (1)	NTMT1	3	DC541	VHL	PEG-Alk (3)	Compound with one PEG unit displayed the highest potency; compounds with linkers of two and four PEG units showed lowered degradation potency	^82^

^a^ The most active in bold

**Table 8.** Experimental methods and cell models applied in development of PROTACs targeting protein methyltransferases.

PROTAC	Target	Cell lines	Cell proliferation/cell cycle/apoptosis	Target degradation	Other assays	Ref
**54** (E7)	EZH2	WSU-DLCL-2, A549, NCI-H1299, Pfeiffer	MTT, optical microscopy	Western blot	*In vitro:* Alpha Screen; thermal shift assay; immunoprecipitation; RT-qPCR	^18^
**55** (YM181) **56** (YM281)	EZH2	22rv1, SU-DHL-2, SU-DHL-4, SU-DHL-6, NCI-BL209, Daudi, Raji, Namalwa, JVM-2, MINO, Jeko-1, primary lymphoma cells extracted from various lymphoma patient samples	MTS; flow cytometry; CellTiter-Glo	Western blot	*In vitro:* immunohistochemistry; immunoprecipitation; Caco-2 cell permeability *In vivo*: Balb/c nude mice (tumour xenograft studies with SU-DHL-6 and Jeko-1 cells) (tumour volume, weight loss	^71^
**57** (MS177)	EZH2	293T, HeLa, MV4;11, RS4;11, MOLM-13, KOPN-8, EOL-1, K562, THP-1, MM.1S, (KO; CRBN−/−)	Cell counter	Western blot	*In vitro*: immunoprecipitation sequencing; Cleavage Under Targets & Release Using Nuclease; co-immunoprecipitation; RNA-seq; Luciferase Reporter Assay; isothermal titration calorimetry; EZH2 methyltransferase assay; ubiquitination assay *In vivo:* Swiss albino mice (pharmacokinetics, complete blood counting) NOD-SCID mice (patient-derived xenograft (PDX) models (intra-tumour/intra-plasma drug concentration analysis)	^72^
**58** (U3i)	EZH2	MDA-MB-231, MDA-MB-468, BT549, MCF7, MCF10A, LO2, HK-2, HL-60, MV4-11	CCK-8; MTT; thymine analogue incorporation; GreenNuc™ caspase-3; Annexin V-PE; flow cytometry	Western blot; mass spectrometry	*In vitro:* surface plasmon resonance; JC-1 assay	^73^
**59** (MS8815)	EZH2	SUM159, MDA-MB-468, MDA-MB-453, BT549, patient primary cell line 515a	CCK-8	Western blot	*In vitro:* EZH2/EZH1 methyltransferase inhibition and selectivity *In vivo:* Swiss Albino mice (pharmacokinetics)	^74^
**60** (1) **61** (2)	EED	Karpas442, G401, NCi’-H1299, MDA-MB-231, DLD1, HCC2218, IMR32, LNCAP, MCF7, MDA-MB-453, CHp134	CellTiter-Glo	Western blot; mass spectrometry	*In vitro:* surface plasmon resonance; methyltransferase assay (Mtase-Glo); TR-FRET ternary complex formation; immunoprecipitation	^75^
**62** (UNC6852)	EED	HeLa, DB, Pfeiffer, 293T	Cell counter	Western blot; mass spectrometry	*In vitro:* TR-FRET ternary complex formation	^76^
**63** (UNC7700)	EED (EZH2 indirectly)	DB, MDA-MB-468, HeLa		Western blot; mass spectrometry	*In vitro:* TR-FRET ternary complex formation	^77^
**64 (**MS147)	EED	K562, KARPAS-422, NCI-H1299, MDA-MB-231, 786-O	CCK-8	Western blot	*In vitro*: isothermal titration calorimetry; RT-qPCR; immunoprecipitation; activity of methyltransferases and thermal shift assay	^78^
**65** (MS9715)	NSD3	EOL-1, RS4;11, MOLM-13, K562, MM.1S, 293FT	Cell counter	Western blot; mass spectrometry	*In vitro:* colony formation assay, immunoprecipitation; ubiquitination; RT-qPCR; isothermal titration calorimetry; RNA-seq	^79^
**66** (SYL2158)	NSD3	NCI–H1703, H520, NCI–H1299, NCI–H358, NCI–H2122, HCC827, EBC-1, SK-MES-1	CCK-8; flow cytometry	Western blot	*In vitro:* RNA-seq; RT-qPCR; colony formation assay *In vivo:* nude mice (tumour xenograft studies with A549 cells; NSD3 expression)	^80^
**67** (MS159)	NSD2	NCI-H929, KMS11, 293FT (CRBN^−/−^	Cell counter	Western blot	*In vitro:* isothermal titration calorimetry; methyltransferase inhibition and selectivity *In vivo*: Swiss Albino mice (pharmacokinetics)	^81^
**68** (MS4322)	PRMT5	MCF7, HeLa, A549, A172, Jurkat	CellTiter-Glo	Western blot	*In vitro:* PRMT5/MEP50 methyltransferase inhibition *In vivo*: Swiss albino mice (PK)	^17^
**69** (1)	NTMT1	HTC116, HT29	WST-8; CyQUANT-GR; flow cytometry	Western blot; NTMT1 cellular degradation; mass spectrometry	*In vitro*: HPLC inhibition assay; tumour spheroid assay	^82^

In 2021 GSK126 and EPZ6438-based EZH2-targeted PROTACs were reported by Liu et al. The most active E7 molecule (**54**), which contained a tazemetostat-based moiety not only degraded EZH2, but also EED, SUZ12, and RBBP4/7 subunits. **54** also decreased H3K27me2/3 levels in various cancer cells and demonstrated antiproliferative activities dependent on the enzymatic and nonenzymatic activities of PRC2. Interestingly, treatment of WSU-DLCL-2 cells with **54** resulted in elevated ubiquitination levels not only of EZH2, but also EED and SUZ12, although direct binding only to EZH2 was observed in cells^18^.

In the same year, EZH2-targeted PROTAC were developed by Tu et al.^71^ They synthesized two series, one recruiting VHL and the other – CRBN E3 ligase, combined with EPZ6438. None of the compounds linked to the thalidomide revealed a promising EZH2 degradation capacity. VHL-targeting YM181 (**55)** and YM281 (**56**) potently degraded EZH2 with substantial selectivity over EZH1 and inhibited proliferation in both diffuse large B-cell lymphoma (DLBCL) and other lymphoma subtypes outperforming EPZ6438 that was only effective against DLBCL. In lymphoma xenografts as well as patient-derived primary lymphoma cells, **55** and **56** demonstrated promising antitumor effects.

MS177 (**57**), a potent EZH2-targeted PROTAC, which consisted of pomalidomide conjugated to C24 via a short one oxygen atom-containing linker, was designed to suppress EZH2's multiple activities. EZH2 recruits and binds also non-PRC2 factors to control gene expression during oncogenesis. As a result, it is possible that the noncanonical activities of EZH2 will not be suppressed by the current EZH2 inhibitors because they only inhibit EZH2’s catalytic activity. The authors showed that in acute leukemias, EZH2 has extra noncanonical capabilities to form interaction with MYC and P300. In multiple MLL1-rearranged leukaemia cells, **57** depleted EZH2, EED and SUZ12 and demonstrated the significant anti-tumour effect in a variety of cancer lines and patient-derived models as well as in multiple tumour cell line xenografted and PDX models^72^. The same group further showed that in multiple myeloma immune response genes were also reactivated by **57**-induced degradation of both canonical EZH2-PRC2 and noncanonical EZH2-MYC complexes^83^. Additionally, the same group recently reported that EZH2 contains a cryptic transactivation domain (TAD) that not only mediates direct interactions with P300 and MYC but also with another member of the nuclear receptor superfamily, androgen receptor (AR) and AR spliced variation 7 (AR-V7), a constitutively active AR variant enriched in advanced castration-resistant prostate cancer (CRPC). **57** depleted both canonical (EZH2:PRC2) and non-canonical (EZH2TAD:AR/AR-V7:co-activators) EZH2 complexes in prostate cancer cells, evoking significantly more robust antitumor effects than EZH2 catalytic inhibitors^84^.

Wang et al. designed a series of degraders based on EZH2 inhibitors, UNC1999, GSK126 and EPZ6438 connected with VHL and CRBN ligands through various linkers of different composition and length. The most active U3i (**58)** was formed by combining UNC1999 and CRBN, linked via PEG (*n* = 5) and the carbon chain (*m* = 7), which were connected by a triazole ring. In triple-negative breast cancer cells (MDA-MB-231 and MDA-MB-468), **58** promoted degradation of EZH2, SUZ12 and EED, inhibited proliferation, induced apoptosis. **58** appeared to be an effective and selective antitumor agent causing little damage to normal cells^73^.

Triple-negative breast cancer cells (TNBC) were also targeted by a series of PROTACs developed by Dale et al. who synthesized EZH2 degraders by connecting EPZ6438 to VHL. MS8815 (**59**) with a seven-methylene linker efficiently degraded EZH2 in MDA-MB-453 cells and exhibited anti-proliferative activity against a variety of TNBC cell lines and the primary patient TNBC ductal adenocarcinoma cells with nanomolar potency. Other PRC2 core components EED and SUZ12 protein levels also decreased with observable EZH2 degradation.

Therefore, **59** was shown to solve the drawback of conventional catalytic inhibitors, which solely target EZH2's catalytic functional domain in TNBC cells^74^.

### PROTACs targeting lysine methyltransferases: EZH2 through EED

Only a few studies have mentioned the phenomenon of PROTACs degrading proteins indirectly, e.g. as the components of a protein complex. Hsu et al. as well as Potjewyd et al. recently reported the discovery of EED-targeted PROTACs, which in fact caused the degradation not only of EED, but also EZH2, and SUZ12, components of the PRC2 complex^75^^,^^76^.

Hsu et al. derivatized an EED inhibitor to hijack VHL ligase. PROTACs 1 (**60**) and 2 (**61**) caused the degradation of EED followed by other PRC2 components, EZH2 and SUZ12. Only in the presence of **60** and **61**, ternary complex formation took place. EED degradation caused by PROTAC was probably responsible for EZH2 and SUZ12 degradation. Alternately, it’s possible that after the ternary complex was formed, PROTAC-mediated ubiquitin attachment could occur at exposed lysine residues in EZH2 or SUZ12 that were close to the EED binding site. A global proteomics analysis revealed that the only proteins which level significantly decreased 24 h after treatment with **60** and **61** were EED, EZH2, and SUZ12. The growth of EZH2 mutant DLBCL cell line Karpas422 and EZH2 WT rhabdoid cancer cell line was potently inhibited^75^.

UNC6852 (**62**) is an example of PROTACs that target EED and is derived from EED226 ligand binding the WD40 domain of EED coupled with VH032-amine, VHL ligand. The most active PROTAC, **62**, had a short three methylene alkyl linker. Adding a fourth methylene group (UNC6853) was enough to significantly slow down EED degradation This confirms that this system is sensitive to the spatial proximity and orientation of the two ligands. **62** degraded both wild-type and mutant EZH2 in HeLa and DLBCL cells with EZH2 gain-of-function mutations in addition to having anti-proliferative properties. Overall, similar to **60** and **61** developed by Hsu et al.,^75^ **62** exerted high selectivity in PRC2 complex degradation within the proteome^76^.

The optimization of **62** led to the development of UNC7700 (**63**), a second-generation EED-targeted PRC2 degrader using a *cis*-cyclobutane linker instead of the propyl linker of **62**^77^. In diffuse large B-cell lymphoma DB cells, **63** degraded EED 15-fold more potently than **62** and could efficiently degrade the main PRC2 components. **63** also decreased H3K27me3 levels more effectively and had a stronger anti-proliferative effect than **62**. Bashore et al. also investigated the impacts of *cis*- and *trans-*cyclobutane linkers on binary target engagement, cell permeability, and ternary complex formation to better understand the differences in degradation efficiency. The PRC2 degradation profile of **63**, which has a *cis*-cyclobutane linker, was different from that of its closely related *trans*-isomer, UNC7698. Particularly, UNC7698 was more selective for EED, whereas **63** potently destroyed both EZH2 and EED. The *cis*- and *trans*-cyclobutane isomers were found to have different levels of cellular permeability with the *cis*-cyclobutane isomer showing noticeably higher permeability. According to molecular dynamics, **63** mostly existed in a folded state with a smaller surface area exposed to solvent, perhaps due to intramolecular hydrogen bonding, while UNC7698 continued to exist in an extended state that may result in decreased permeability.

PROTACs targeting EED were also developed by the same group to degrade components of Polycomb repressive complex 1 (PRC1). The catalytic core, the E3 ubiquitin ligase RING1A/B responsible for initiating the monoubiquitylation of histone H2A at lysine 119 (H2AK119ub1), interacts with one of six Polycomb group RING finger (PCGF1-6) proteins. PCGFs and RING1A/B are overexpressed in many different types of cancer, including breast, lung, and leukaemia progression. Therefore, targeting PRC1 core components could provide a potential therapeutic approach for treating cancers. Based on the fact that EED interacts with PRC1 core components in addition to being one of PRC2's core components, Park et al. developed PROTACs that employed EED226 binding EED, an interacting protein partner of BMI1 and RING1B, to recruit BMI1 and RING1B to the E3 ligase VHL for ubiquitination and degradation. The most active EED-binding MS147 (**64**) preferentially degraded PRC1 core components, PCGF4 (also known as BMI1) and RING1B, over EED in K562, MDA-MB-231, NCI-H1299 cells and KARPAS-422 cells. As a result, the H2AK119ub mark, which is catalyzed by PRC1, was effectively reduced by **64** without affecting the H3K27me3 mark, which was catalyzed by PRC2. **64** was able to trigger the formation of the BMI1-RING1B-EED-MS147-VHL complex, bringing BMI1 and RING1B closer to VHL for ubiquitination and degradation. While **64**, the BMI1 and RING1B degrader, and PROTAC 2, the EED/PRC2 degrader, share a similar VHL ligand and EED binder, **64** and PROTAC 2 differ in their linkers. The difference in their degradation profiles is most likely due to this linker difference^78^.

### PROTACs targeting lysine methyltransferases: NSD2 and NSD3

The Nuclear Receptor-Binding SET Domain (NSD), which catalyzes the mono- and dimethylation of histone H3 lysine 36 (H3K36me2), is another lysine methyltransferase of interest since it acts as an oncoprotein. Overexpression and mutations of the three NSD family members (NSD1, NSD2, and NSD3) have been linked to several types of cancers, including lung cancer, breast cancer, prostate cancer, acute myeloid leukaemia, acute lymphoblastic leukaemia, and multiple myeloma. For the purpose of developing NSD inhibitors and degraders, it has been demonstrated that focusing on NSDs outside of the catalytic domain is an effective strategy. Two PWWP domains, one for histone methyl-lysine recognition and the other for interaction with DNA and histone, are found in NSD proteins in addition to the SET domain. Xu et al. linked BI-9321, a selective and potent antagonist of NSD3-PWWP1, with a VHL binder VHL1-Me to create MS9715 PROTAC (**65**). NSD3 was the only protein that was significantly downregulated. **65** degraded NSD3 and MYC since NSD3 functions as an adaptor that is linked to MYC as well as effectively suppressed gene expression programs associated with NSD3 and MYC, similar to the effects of the CRISPR-Cas9-mediated knockout of NSD3. **64** also effectively inhibited the proliferation of multiple cell models of haematologic cancer cells^79^.

In the same year Sun et al. also utilized BI-9321 to synthesize a set of NSD3-targetd PROTACs. The linker underwent extensive modification and several potent NSD3 degraders have been identified. In NCI-H1703 and A549 cells, SYL2158 (**66**) induced NSD3 degradation and effectively inhibited H3K36 methylation, the expression of NSD3-associated genes, and clone formation in lung cancer cells, in addition to possessing good selectivity over the other two NSD proteins (NSD1 and NSD2). In the A549 xenograft tumour mouse model, a single dose of **66** successfully induced the degradation of NSD3^80^.

The first-in-class NSD2 PROTACs were described by Meng et al. The most active degrader MS159 (**67**) connects the selective NSD2-PWWP1 antagonist UNC6934 which disrupts the PPIs between the NSD2-PWWP1 to a CRBN E3 ligase ligand. This PROTAC inhibited proliferation of KMS11 and H929 multiple myeloma cells much more effectively than UNC6934, indicating that pharmacological degradation of NSD2 and IKZF1/3 is a superior therapeutic strategy to pharmacological antagonism of the NSD2-PWWP1 and chromatin PPIs. Moreover, **67** was bioavailable in a mice model^81^.

### PROTACs targeting protein arginine methyltransferases: PRMT5

The PRMT family, particularly PRMT5, has been extensively researched in cancer therapy due to its implication in the regulation of cancer-related processes such as transcription, DNA repair, and RNA metabolism. A first-in-class PRMT5 PROTAC was created by Shen et al. by combining the PRMT5 competitive inhibitor EPZ015666 with a VHL ligand, (*S,R,S*)-AHPC-Me. Longer PEG linkers resulted in degraders that were able to reduce cellular PRMT5 protein levels to a greater extent, according to SAR studies. Following reports that the VHL binder VHL-2 produces more effective degraders, VHL-1 was substituted in the synthesis of MS4322 (**68**), which was found to be highly selective for PRMT5 in a global proteomics study and demonstrated antiproliferative activity in MCF7, Hela, A549, A172, and Jurkat cells and promising pharmacokinetic properties in mice. Only PRMT5, its binding partner WDR77 and an unrelated protein called AGRN had their levels decreased^17^.

### PROTACs targeting N-terminal methyltransferases: NTMT1

Protein N-terminal methylation entails adding up to three methyl groups to the protein’s free amino group at the N-terminus. Except for proline dimethylation, the fully methylated state is trimethylation, which can result in a pH-insensitive positive charge on the protein N-termini. NTM regulates protein-protein (PPI) and protein-DNA interactions and dysregulation of NTMT1 has been linked to a variety of diseases, including malignant melanoma and colorectal, breast, lung, and brain cancers. NTMT1 was found to be significantly overexpressed in colorectal cancer patients, ranking in the top 1% of all proteins undergoing expression level changes. The first-in-class NTMT1 PROTAC was developed by connecting a NTMT1 peptidomimetic inhibitor DC541 to a VHL E3 ligand via a short, three atom long linker. In colorectal carcinoma cell lines HCT116 and HT, a degrader 1 (**69**) reduced NTMT1 protein levels effectively and selectively. Although **69** had marginal cytotoxicity, it displayed anti-proliferative activity in a 2D and 3D culture environment as a result of HCT116's cell calreticulin, an immunogenic cell death signal protein that is known to elicit an antitumor immune response and is clinically linked to a high survival rate of patients with colorectal cancer upon its upregulation^82^.

## ADP-ribosylation

### ADP-ribosyltransferases

**Figure 9.**
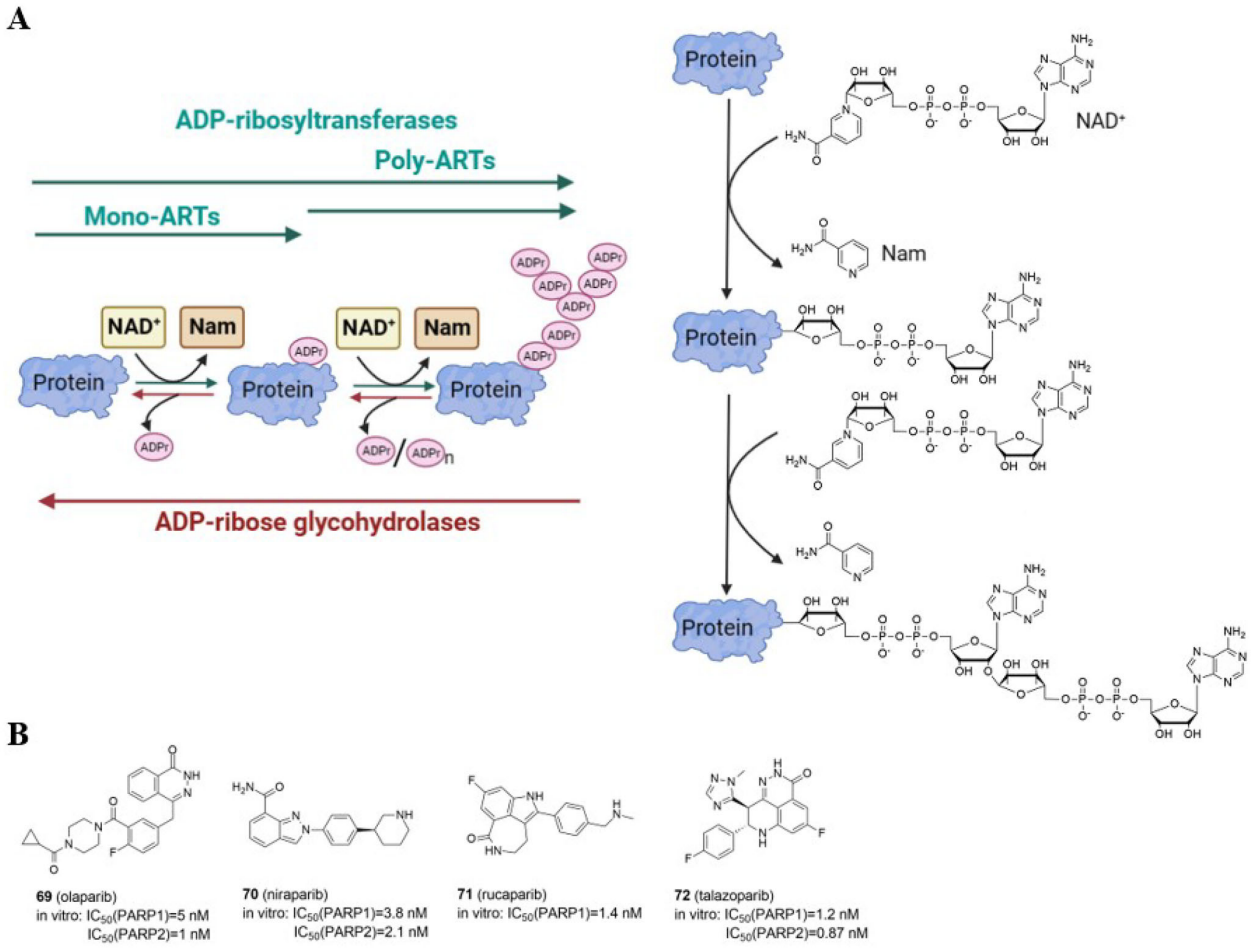
Protein ADP-ribosylation catalyzed by ADP-ribosyltransferases (ARTs) with enzymatic activity of mono- and poly-ADP-ribosylation (MARylation and PARylation, respectively) (A) (created in BioRender.com). Structure of four PARP inhibitors approved by FDA (B).

ADP-ribosylation is a reversible PTM in which the ADP-ribose moiety is transferred from NAD^+^ onto target proteins by ADP-ribosyltransferases (ARTs) (Figure 9(A)).

Human PARPs (previously poly-ADP-ribose polymerases) and tankyrases (TNKS) are members of the ART family divided into mono-ARTs and poly-ARTs (Table 9). Poly-ARTs (PARP1-2 and TNKS1-2) catalyze the covalent attachment of multiple ADP-ribose units, resulting in poly-ADP-ribosylation (called PARylation), whereas mono-ARTs (PARP3-4, −6–8, −10–12, and −14–16) do not generate polymers but instead catalyze the addition of a single ADP-ribose unit (MARylation). Glu, Asp, Ser, Thr, Tyr, Arg, Lys, His, and Cys are currently known to be ADP-ribosylated amino acids. ARTs can be also classified based on the characterized domains. The first group, which includes PARP1/2/3, by binding to damaged DNA via the N-terminal binding domain, mediates DNA damage repair. Tankyrases such as TNKS1 and TNKS2 capture substrates via tankyrase-binding peptide motifs. The third subfamily, which includes PARP7/12/13, is a member of the CysCysCysHis zinc finger family and can bind viral RNA. The fourth subclass includes PARP9, PARP14, and PARP15, with macrodomains that can mediate protein migration to PARylation and MARylation sites. PARPs 4, 6, 8, 10, 11, 16 and 17 are unclassified^85^.

**Table 9.** Categorisation of ADP-ribosyltransferases (ARTs).

ADP-ribosyltransferases (ARTs)					
ARTD Family			ARTC family		
Preffered name	Aliases	Main activity	Preffered name	Aliases	Main activity
**PARP1** ^a^	ARTD1, PARS	PARylation	ART1	ARTC1	MARylation
**PARP2**	ARTD2	PARylation	ART2	ARTC2	MARylation
PARP3	ARTD3	MARylation	ART3	ARTC3	Inactive
PARP4	ARTD4	MARylation	ART4	ARTC4	Inactive
TNKS1	ARTD5, PARP5a	PARylation	ART5	ARTC5	MARylation
TNKS2	ARTD6, PARP5b	PARylation			
PARP6	ARTD17	MARylation			
PARP7	ARTD14	MARylation			
PARP8	ARTD16	MARylation			
PARP9	ARTD9	MARylation			
PARP10	ARTD10	MARylation			
PARP11	ARTD11	MARylation			
PARP12	ARTD12	MARylation			
PARP13	ARTD13	Inactive			
**PARP14**	ARTD8	MARylation			
PARP15	ARTD7	MARylation			
PARP16	ARTD15	MARylation			

^a^Enzymes for which PROTACs have been developed are marked in bold

DNA single-strand breaks (SSBs) that are repaired but do not progress to double-strand breaks (DSBs) activate PARP1–3. PARP1 is the most abundant and well-studied PARP, and it is highly expressed in cancer cells. Overexpression of PARP1 has been demonstrated e.g. in breast, lung cancers and melanoma^86^. PARP1 can bind to DNA damage sites, causing DNA repair proteins to be recruited to restore DNA breaks and maintain genomic stability. BRCA1/2 are essential enzymes in the repair of DNA DSBs by promoting the homologous recombination repair (HRR), and as BRCA deficiency is a common feature in multiple cancer cells, including breast and ovarian cancers, PARP inhibitors (PARPi) are used in the treatment of oncological diseases. Many PARPi small molecule compounds have been tested at various stages of clinical trials. Olaparib (**70**)^87^, niraparib (**71**)^88^, rucaparib (**72**)^89^, and talazoparib (**73**)^90^ (Figure 9(B)) are currently approved by FDA for the treatment of patients with a range of cancers (e.g. advanced ovarian, metastatic breast, prostate). Since PARPi structurally mimic NAD^+^, PARPi’s mechanism of action leads to the formation of the PARP1-PARPi-DNA complex, which prevents DNA replication. The resulting triplet is more dangerous than just an unfixed SSB^91^. Most of the current PARP inhibitors target either PARP1 and/or PARP2, and are highly effective against BRCA-mutant cancers but have significant side effects. Notably, inhibition of PARP2 has been linked to hematological toxicity^92^.

### PROTACs targeting ADP-ribosyltransferases: PARP1

**Figure 10.**
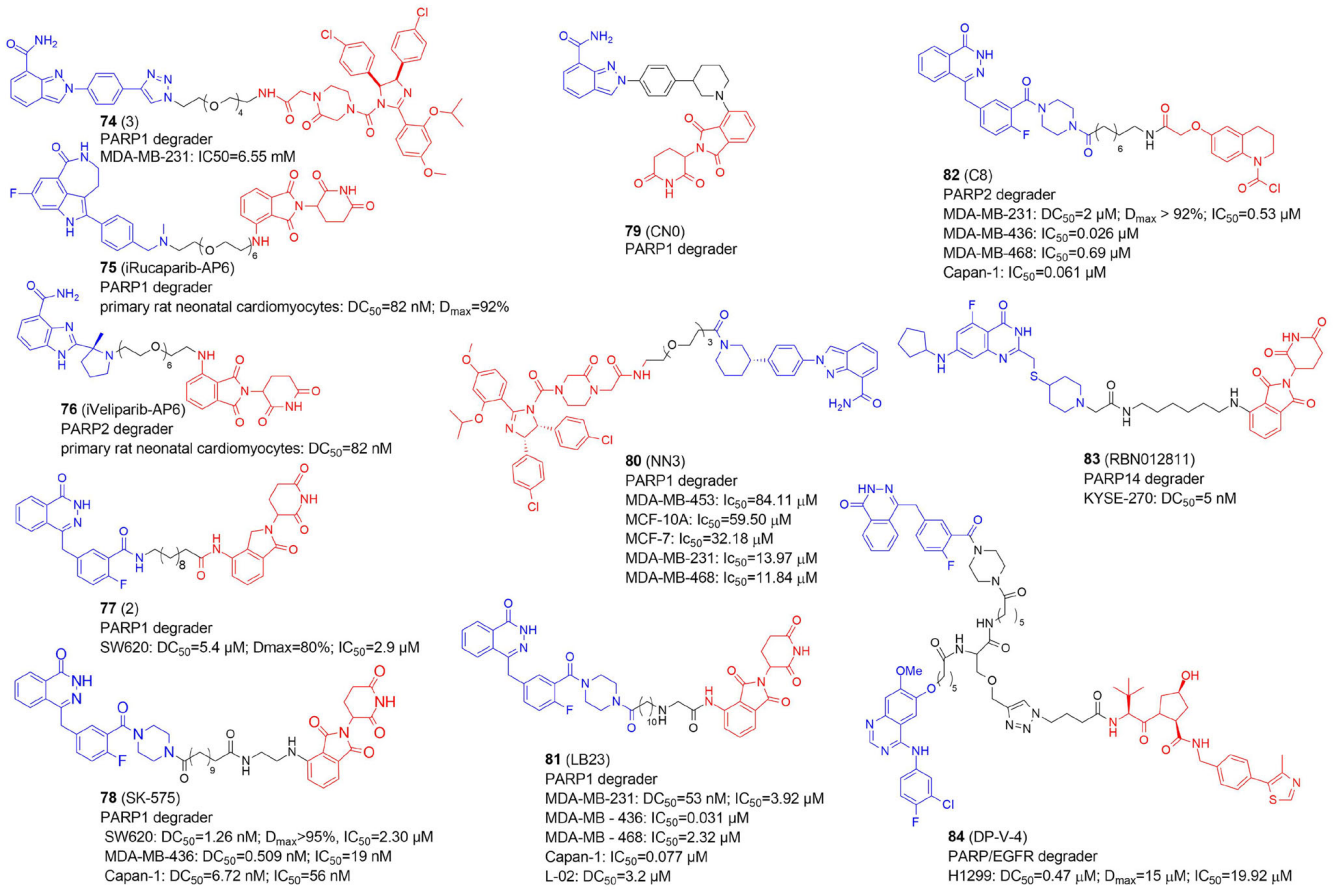
Structures and activities of PROTACs targeting ADP-ribosyltransferases.

In recent years, several PROTACs degrading PARP1 have been reported (Figure 10, Tables 10 and 11). In 2019 Zhao et al. published the first PARP1-targeted PROTAC using a nutlin-3 derivative and niraparib^16^. By linking nutlin, the MDM2 ligand, and the PARP1/2 inhibitor niraparib, compound 3 (**74**) was found to selectively induce significant PARP1 degradation and cell apoptosis in MDA-MB-231 cells following a comprehensive degradation screening in several TNBC cell lines. In addition, **74** exhibited no cytotoxicity in normal cells and was five times more potent than PARP1 inhibitors (niraparib, olaparib, and veliparib).

**Table 10.** Components and structural details of ART-PROTACs.

PROTAC	Target	# of analogs	Original inhibitor	E3 ligase ligands^a^	Type of linker (# of analogs)	Comments	Ref
**74** (3)	PARP1	5	Niraparib Olaparib	**MDM2** CRBN VHL	Alk-PEG (5)		^16^
**75** (iRucaparib-AP6) **76** (iVeliparib-AP6)	PARP1 PARP2	27	**Rucaparib** **Veliparib**, Olapararib, Niraparib	**CRBN,** VHL	PEG (16) Alk-PEG (2) Triazole-PEG (9)	Only compounds with either solely PEG linkers or PEGs combined via triazole with alkyl linker (4C) were active; shortening the alkyl-triazole-PEG linker to less than 3PEG units, or changing triazole into amide, or switching the linker conjugation site abolished PARP1 degradation activity; VHL-Rucaparib analogs showed lowered PARP1 degradation	^93^
**77** (2)	PARP1	3	Olaparib	**CRBN**	Alk (3)	Compounds with linkers 10 and 11 carbon long degraded PARP1 protein more strongly than compound with 8 carbon atom-linker	^94^
**78** (SK-575)	PARP1	27	Olaparib	**CRBN**, VHL	Alk (24) PEG (3)	Optimal linker: dodecarboxylic acid (PROTAC of similar potency to olaparib in cell growth; adding or deleting one methylene unit led to less potent PROTACs	^95^
**79** (CN0)	PARP1	7	Niraparib	**CRBN**	Alk (1) PEG (6)	CN0 with no extra PEG linker degrades PARP1 effectively. The aromatic ring or aliphatic ring of the PARP1 inhibitor itself can act as a linker to form an effective PARP1 degrader	^95^
**80** (NN3)	PARP1	1	Niraparib	**MDM2**	PEG (1)	–	^96^
**81** (LB23)	PARP1	29	Olaparib	**CRBN**	Alk (19) PEG (6) PEG-triazole (3) PEG-trazole-Alk (1)	Part of the linker of LB23 constitute a 2-amino-acetamide group.It might be responsible for selective degradation of target protein only in tumour cells, while in normal cells there might be an enzyme that catalyzes the degradation of the 2-amino-acetamide group of LB23	^97^
**82** (C8)	PARP2	29	Olaparib	**DCAF16 (KBO2) covalent ligand**	Alk (20) PEG-triazole-Alk (1) PEG (8)	Carbonyl group directly linked to nitrogen of piperazine in Olaparib was crucial for activity; alkyl or alkyl-triazole-PEG analogs were more potent than PEG analogs; optimal length of alkyl linker was 10-12, while shorter (C4-C9) or longer (C13) analogs showed reduced antiproliferative and degradation activity towards PARP2 in MDA-MB-231 cells	^98^
**83** (RBN012811)	PARP14	1	RBN012042	CRBN	Alk (1)	–	^99^
**84** (DP-V-4)	PARP, EGFR	12	Olapari Gefitinib	CRBN **VHL**	Star-like (8) Other (2) Alk-tiazole (2)	The star-like linker derived from biocompatible natural amino acids, tyrosine and serine, were used to connect three different small molecules, two inhibitors and one E3 ligase ligand	^100^

^a^ The most active in bold

**Table 11.** Experimental methods and cell models applied in development of PROTACs targeting ADP-ribosyltransferases.

PROTAC	Target	Cell lines	Cell proliferation/cell cycle/apoptosis	Target degradation	Other assays	Ref
**74** (3)	PARP1	MDA-MB-231, MCF10A	CCK-8; Annexin-V/PI; flow cytometry	Western blot		^16^
**75** (iRucaparib-AP6) **76** (iVeliparib-AP6)	PARP1 PARP2	Primary rat neonatal cardiomycytes, HeLa, BT-549,	Flow cytometry	Western blot; mass spectrometry		^93^
**77** (2)	PARP-1	SW620	CCK8; flow cytometry	Western blot	*In vitro*: migration scratch assay	^94^
**78** (SK-575)	PARP-1	22RV1, HCC1937, PC-3, MDA-MB-468, LNCaP, MDA-MB-231, SW620, Capan-1	CKK-8	Western blot	*In vivo*: BALB/c nude mice (tumour xenograft studies with SW620 or Capan-1 cells) (H&E staining)	^95^
**79** (CN0)	PAPR1	MDA-MB-231, MCF10A, 4T1	MTT	Western blot	*In vitro*: cellular thermal shift assay; RT-qPCR *In vivo:* mice (allograft 4T1 tumours were treated with CN0) (tumour volume and immunohistochemistry)	^95^
**80** (NN3)	PARP1	MCF7, MDA-MB-231, MDA-MB-453, MDA-MB-468, MCF10A, HCC1937	MTT; flow cytometry	Western blot	*In vitro:* cellular thermal shift assay; colony formation; RT-qPCR; GSH assay; ROS and MDA assay *In vivo*: BALB/c nude mice (tumour xenograft studies with MDA-MB-231) (tumour volume and immunohistochemistry)	^96^
**81** (LB23)	PARP-1	MDA-MB-436, MDA-MB-231, MDA-MB-468, Capan-1	MTT; flow cytometry	Western blot	*In vitro*: colony formation assay	^97^
**82** (C8)	PARP-2	MDA-MB-436, MDA-MB-231, MDA-MB-468, Capan-1	MTT; flow cytometry	Western blot	*In vitro*: colony formation assay *In vivo*: BALB/c nude mice (tumour xenograft studies with MDA-MB-231 cells) (tumour volume)	^98^
**83** (RBN012811)	PARP14	KYSE-270, human PBMC, 293T		Western blot		^99^
**84** (DP-V-4)	PARP, EGFR	SW1990, H1299	CKK-8	Western blot		^100^

In 2019 Wang et al. also reported the development of PARP1 degraders. In primary rat neonatal cardiomyocytes, iRucaparib-AP6 (**75**) based on an FDA-approved PARP1/2/3 inhibitor rucaparib caused strong PARP1 degradation at doses as low as 50 nM. By reducing the level of PARP1, **75** rescued muscle cells and primary cardiomyocytes against the energy crisis and cell death caused by DNA damage. **75** was shown to be a suitable method for the treatment of malignancies and other disorders induced by PARP1 hyperactivation by blocking both the catalytic and scaffolding activities of PARP1. The **75** warhead was also replaced with therapeutically relevant PARP1 inhibitors such as niraparib, olaparib, and veliparib. Of note, iRucaparib-AP6 and iVeliparib-AP6 (**76**) with the same CRBN ligand and linker, but with different POI ligand, show degradation selectivity of PARP1 or PARP2, respectively^93^.

In 2020 Zhang et al. conjugated olaparib, the FDA-approved PARP1/2 inhibitor and lenalidomide to synthesise PROTACs tested in colorectal cancer SW620 cells^94^. Compound **2** (**77**) appeared to be an efficient PARP1 degrader inhibiting SW620 cell growth and induced cell apoptosis.

In the same year, Cao et al. reported the compound SK-575 (**78**), which also reduced PARP1 levels in SW620, MDA-MB-436, and Capan-1 cells at low picomolar concentrations^95^. When used alone or in combination with cytotoxic agents like temozolomide and cisplatin, **78** inhibited tumour growth in mice.

The CN0 (**79**) prepared and tested by Lin et al. also proved to effectively and selectively degrade the PARP1 protein in MDA-MB-231 cells, with no effect on the level of PARP2 and PARP3^96^. Interestingly, the superior degrader **79** did not contain any linker. **79** suppressed DNA damage repair when combined with daunorubicin, resulting in a highly effective accumulation of cytosolic DNA fragments caused by unrepaired DNA lesions.

PARP1 was also degraded in MDA-MB-231 and MCF7 cells by Li et al. using PROTAC NN3 (**80**) containing niraparib tethered to nutlin-3a, an MDM2 ligand^97^. **80** demonstrated substantial antiproliferative action and minimal toxicity in TNBC cells, with a novel mechanism including ferroptosis activation via SLC7A11 pathway downregulation in p53^+^ cells.

In 2022, Pu et al. developed a series of PROTACs with various linkers by utilizing olaparib and thalidomide/pomalidomide. The compound LB23 (**81**), with nearly 60-fold tumour-selectivity effectively degraded PARP1 in cancer cells and inhibited MDA-MB-231 cell proliferation by halting the cell cycle in the G2/M phases^98^.

### PROTACs targeting ADP-ribosyltransferases: PARP2

PARP1 and PARP2 have distinct roles and mechanisms in tumour development, despite sharing a similar catalytic domain. Single PARP2-deficiency was shown to inhibit tumour progression while dual PARP1/PARP2-deficiency promotes tumour growth in T cells. In addition, only PARP2 controls haematopoiesis and T-cell development. This suggests that selective inhibition of PARP2 may offer new hope for the effects of antitumor drugs. However, there aren’t many reports yet of PARP2's selective degradation. Pu et al. developed PROTACs that selectively degraded the PARP2 protein. They decided to use a covalent ligand KB02 of a DCAF16 ligase (DDB1 And CUL4 Associated Factor 16) and olaparib with linkers of various lengths and compositions. Among synthesized compounds, the C8 (**82**) turned out to degrade PARP2 in MDA-MB-231 breast cancer cells the most effectively and selectively. **82** also effectively inhibited the tumour growth in BRAC2 negative TNBC xenograft model^101^. Of note, **81** exhibited PARP1 selectivity having a different E3 ligase ligand but the same POI ligand and very similar linker as **82**.

### PROTACs targeting ADP-ribosyltransferases: PARP14

Several PROTACs for poly-ARTs have already been reported as potent and efficacious in the treatment of cancer but only one degrader for mono-ART PARP14 has been reported so far by Ribon Therapeutics^99^. The selective PARP14 inhibitor RBN12042 (selectively inhibiting PARP14 more than 100-fold over all other human ART enzymes) was tethered to thalidomide via a suitable linker to produce degrader RBN012811 (**83**). **83** was evaluated in KYSE-270 cells (human oesophageal squamous cell carcinoma), and it was found to degrade endogenous PARP14 without changing the levels of the other ARTs. PARP14 in MDA-MB-231 and JJN-3 (lymphoma cell line) was also shown to be degraded, as well in HEK293T and human macrophages.

### PROTACs targeting ADP-ribosyltransferases: dual PARP and EGFR degradation

In 2021, Zheng et al. unveiled the dual PROTAC successfully degrading both PARP and the epidermal growth factor receptor (EGFR) in the H1299 cell line. The DP-V-4 compound (**84)** consisted of an E3 ligase ligand, a star-like linker, and two independent inhibitors linked thereto^100^. PARP degradation activity was slightly higher than that of EGFR. Its antiproliferative activity in H1299 tumour cells measured as IC_50_ was 19.92 µM, which was between that of the EGFR inhibitor Gefitinib (IC_50_ = 6.56 µM) and that of the PARP inhibitor olaparib (IC_50_ = 35.93 µM). The lower antiproliferative activity of **84** may be due to the degrader’s higher molecular weight, which causes poor solubility and cell permeability.

## Ubiquitination

E3 ligases playing a critical role in determining the type of ubiquitinated substrate have been classified into three groups based on their catalytic structure, namely the RING family, the HECT (**h**omologous to **E**6AP **C**-**T**erminus) family, and the RBR (RING-between-RING) family (Table 12). Ubiquitination is catalyzed by the HECT and RBR families in two steps. The catalytic transfer of Ub first occurs from the E2 to the E3, then from the E3 to the substrate. Instead of using an intermediate E3-Ub, the RING family mediates the direct transfer of ubiquitin from the E2 to the substrate^6^.

**Table 12.** Categorisation of E3 ligases with the selected members of each family.

E3 ligases					
RING family			HECT family		
Name	Role	Selected targets	Name	Role	Selected targets
APC/C	Cell cycle	Aurora, CDC6, CDC20, SKP2	ARF-BP1	DNA damage repair	p53
BRCA1/2	DNA damage repair	H2A, RNA polII, TFIIE, NPM1, CtIP, tubulins, ER-α	Msl2	DNA damage repair	p53
**cIAP** ^a^	TNF, NF-κB signalling	RIP1, TRAF2, NIK	Nedd4	PTEN-AKT signalling	AKT, PTEN
CARP1/2	DNA damage repair	p53			
CHIP	PTEN-AKT signalling	AKT, PTEN, p53	**RBR family**		
**CRBN**	BKCa channel regulation, energy metabolism	SLO1, EIS2, AMPKα, CLC-1, neo-substrates upon IMiD binding			
COP1	DNA damage repair	p53	ARIH1	Translation, anti-tumour immunity	EIF4E2, PD-L1
CUL5	DNA damage repair	p53	CUL9	microtubule dynamics	BIRC5
DCAF16	Regulation of gene expression	SPIN4	RNF144B	DNA damage repair	p53
FBXO1	Cell cycle	CP110, RRM2, E2Fs			
FBXO22		BACH1			
FBXO32	Cell proliferation	c-Myc			
FBXW7	Cell cycle and proliferatiion	Cyclin E, c-Myc, c-Jun			
FBW5	mTORC1 signalling	TSC2			
FBXL12	AMPK signalling	CaMKK2			
**KEAP1**	Oxidative stress	Nrf2			
KLHL22	mTORC1 signalling	DEPDC5			
MDM2	p53 signalling	p53, HIF-1α			
MULAN	AKT	AKT			
PIRH2	DNA damage repair	p53			
RNF14	Wnt/β-catenin signalling	TCF4			
RNF152	mTORC1 signalling	RagA, Rheb			
RNF20/40	NF-κB signalling	H2Bub1			
RNF4	Wnt and Notch signalling pathways	β-catenin, c-Myc, c-Jun, NICD			
RNF8	DNA damage repair	H2A			
RNF43/ZNRF3	Wnt/β-catenin signalling	Fzd1/2/3/4/5/8 and LRP5/6			
RNF85	NF-κB signalling	NF-κB kinase			
RNF135	RLR signalling	RIG-I			
SKP2	mTORC1, AKT, AMPK signalling	RagA, LKB1, AKT, c-Myc, p53, p27kip1, p21			
SYVN1	DNA damage repair	p53			
TRAF2	mTORC1 signalling	mLST8			
TRAF6	AKT, mTORC1 signalling	AKT, mTOR, HIF-1α			
TRIM15	Actin cytoskeleton dynamics	FBLP-1, VASP			
**TRIM24**	DNA damage repair	p53			
TRIM25	RLR signalling	RIG-I			
TRIM28	AMPK signalling	AMPKα			
TRIM3	p53 signalling	p53			
TRIM56	STING signalling	STING			
TRIM40	NF-κB signalling	IKK-γ			
TTC3	AKT	AKT			
**XIAP**	TNF, Wnt/β-catenin, PTEN-AKT signalling	Caspase-3, PTEN, TLE			
β-TRCP	Wnt/β-catenin and NF-κB signalling pathways	β-catenin, p53, Yap, IKB, KRAS, Cyclin D1, WEE1, CDC25, MCL-1, FOXO3, CHD1, c-Myc			
UBE4B	DNA damage repair	p53			
**VHL**	Cell metabolism regulation	HIF-1α			

^a^Enzymes for which PROTACs have been developed are marked in bold

### PROTACs targeting E3 ligase

The involvement of E3 ligases in the regulation of fundamental cellular processes and cancer formation implies that they could be important therapeutic targets. One of the reasons that targeting the ubiquitin-proteasome system is an appealing therapeutic strategy is the efficacy of proteasome inhibitors in cancer treatment. Despite the efficacy of proteasome inhibitors (e.g. bortezomib, ixazomib, and carfilzomib) in the treatment of certain cancers, particularly hematological malignancies, undesirable side effects have been reported, presumably due to non-specific inhibition of proteasome-dependent degradation of many cellular substrates^102^. E1 and E2 enzymes are not currently the target of any approved drugs. There is, however, a class of FDA-approved medications targeting CRBN (Figure 1(C)).

**Figure 11.**
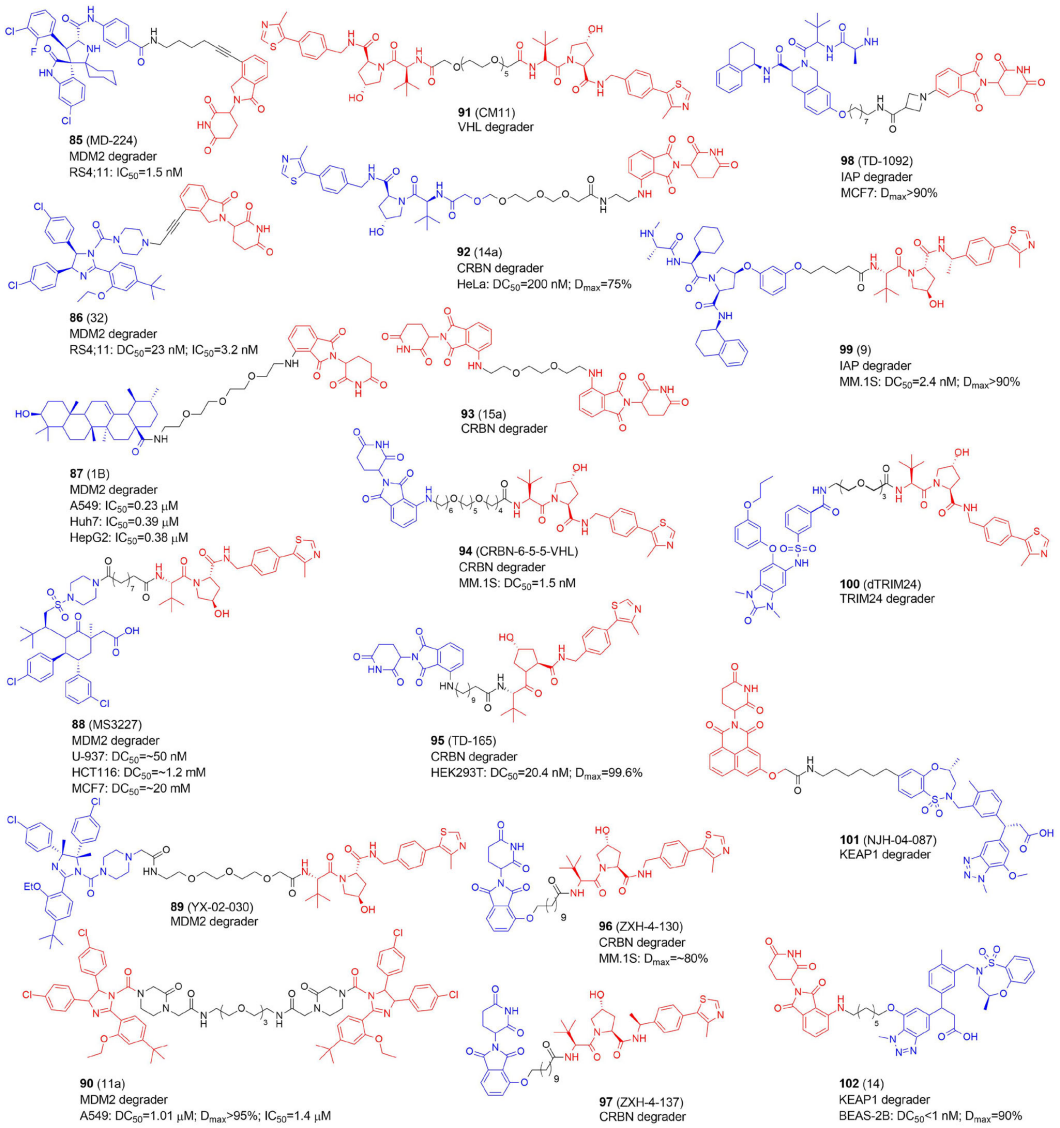
Structures and activities of PROTACs targeting E3 Ligases.

The conventional PROTACs are heterobifunctional compounds designed to cause the breakdown of POI by directly binding its ligand and hijacking E3 ligases. The homobivalent PROTACs aim to dimerise an E3 ligase and then trigger its self-degradation. In this case, the E3 ligase serves as both the neo-substrate and the enzyme at the same time. Homo-PROTACs induce self-degradation by the dual hijacking of the same E3 ligase, as in the case of the VHL-VHL^19^, CRBN-CRBN^103^, and MDM2-MDM2^104^ PROTACs. Another successful method for the depletion of E3 ligases involved using heterodimeric PROTACs to direct various E3 ligases against one another. Examples include CRBN-VHL, MDM2-CRBN^104–106^, MDM2-VHL^107^, VHL-TRIM24^108^, IAP-VHL^109^, IAP-CRBN^109^, KEAP1-CRBN^109^^,^^110^, PROTACs (Figure 11, Tables 13 and 14).

**Table 13.** Components and structural details of E3 Ligases-targeting PROTACs.

PROTAC	Target	# of analogs	Original inhibitor	E3 ligase ligands^a^	Type of linker (# of analogs)	Comments	Ref
**85** (MD-224)	MDM2	19	MI-1061	**CRBN** VHL	Alk (14) PEG (5)	2CH_2_ to 6CH_2_ linkers results in similar potencies in inhibition of cell growth; 7CH_2_ groups is 10 times less potent than 4 CH_2_; linkers with 1, 2, 3 PEG units show similar degradation potency; conversion of two CH_2_ groups into an alkyne group (linker rigidification) increased the potency	^105^
**86** (32)	MDM2	14	RG7112	CRBN	Alk (14)	Optically pure RG7112 (4*S*, 5*R*) **is** more potent than mixture of stereoisomers; degraders with the shortest linker the most efficient	^111^
**87** (1B)	MDM2	6	Ursolic acid	CRBN	PEG (3) PEG-PEG (via ester) (1) PEG-PEG-triazole (1) Alk (1)	Three PEG units demonstrated the best antitumor activity	^106^
**88** MS3227	MDM2	10	AMG 232	VHL	PEG (5) Alk (5)	Compounds with 8CH_2_ and 9CH_2_ -long linkers: the most active; shorter alkyl chains: reduced or none activity; PEG linkers: not effective;	^107^
**89** (YX-02-030)	MDM2	1	RG7112	VHL	Alk- PEG (1)	–	^112^
**90** 11a	MDM2	10	Nutlin-3	MDM2	Alk-PEG (5) Alk (5)	Shorter alkyl linkers: higher potency longer PEG linkers: higher potency	^104^
**91** (CM11)	VHL	9	VH032	VHL	PEG (9)	The most active compounds are symmetrically linked via acetyl group of VH032	^19^
**92 (**14a)	CRBN	13	VH032	VHL	PEG (9) Alk (3) Alk-PEG (1)	The most active CRBN degrader contained a symmetric linker with two terminal carboxylate groups	^113^
**93 (**15a)	CRBN	9	thalidomide	CRBN	PEG (6); polyethers (3)	Analog bearing 2PEG linker was optimal in terms of efficiency of CRBN degradation as well as reduced degradation of neo-substrate, IKZF1; similar properties had 1PEG analog; analogs with longer linkers showed lower CRBN degradation; advantage of a linker attachment via amine bond, advantageous compared with ether or amide linkage with thalidomide; the addition of a linker to the phthalimide part does not interfere with binding of CRBN to neo-substrates	^103^
**94** (CRBN-6-5-5-VHL)	CRBN	8	thalidomide	VHL	PEG (2); polyethers (6)	Short linkers with 8 to 14 atoms less potent in ternary complex formation; longer linkers well tolerated; linker length and lipophilicity rather than polar surface area are activity-determining parameter; preferable clogP >4.5	^114^
**95** (TD-165)	CRBN	8	pomalidomide	VHL	Alk (7) PEG (1)	Only alkyl analogs were active	^115^
**96** (ZXH-4-130) **97** (ZXH-4-137)	CRBN	12	pomalidomide	**VHL** CRBN	Alk (11) PEG (1)	Use of methylated VHL ligand (**96**) results in similar or higher potency PROTAC longer alkyl linkers increase the hydrophobicity and clogP of the whole compound, improving cell permeability	^116^
**98** (TD-1092)	cIAP2, XIAP	11	compound 1	CRBN	Alk (2) Alk-amide of heterocyclic amine (6) Alk-amide_acetic acid analog (2) PEG-amide of heterocyclic amine (1)	Short alkyl and PEG (only one analog tested) linkers not effective in XIAP degradation, in contrast to octyl-3-azetidine carboxylic acid analog	^109^
**99** (9)	IAP	27	CST530	**VHL** CRBN	Alk (7) PEG (6) polyethers (14)	Hetero-PROTACs with C5, C8, and C4 − O − C4 linkers induced the most potent pan-IAP degradation; VHL degradation was less pronounced for compounds containing long and hydrophobic linkers; the most lipophilic IAP-VHL PROTAC showed the lowest IAP degradation, whereas for IAP-CRBN series, high PROTAC lipophilicity led to XIAP-selective depletion	^117^
**100** (dTRIM24)	TRIM24	1	IACS-7e	VHL	PEG	Only one analog and control compound were synthesized	^108^
**101** (NJH-04-087)	KEAP1	2	KI-696	**CRBN** VHL	Alk (2)	KEAP1-VHL PROTACs degraded both VHL and KEAP1 at high concentrations; KEAP1-CRBN PROTACS, NJH-04-087 is selective for KEAP1 degradation	^118^
**102** (14)	KEAP1	12	compound 2	CRBN	Alk (9) PEG (1) Alk-PEG (2)	longer linker length (>7 atoms, 8–13) resulted in a robust KEAP1 degradation compared to shorter linkers; amine linkage instead of acetamide linkage to thalidomide increases stability	^110^

^a^ The most active in bold

**Table 14.** Experimental methods and cell models applied in development of E3 Ligases-targeting PROTACs.

PROTAC	Target	Cell lines	Cell proliferation/cell cycle/apoptosis	Target degradation	Other assays	Ref
**85** (MD-224)	MDM2	RS4;11	WST-8; flow cytometry	Western blot	*In vitro:* fluorescence polarization-based binding assay *In Vivo:* SCID mice (tumour xenograft studies with RS4;11 cells) (tumour volumes and pharmacodynamis)	^105^
**86** (32)	MDM2	RS4;11	MTT; flow cytometry	Western blot	*In vitro:* RT-qPCR	^111^
**87** (1B)	MDM2	A549, Huh7, HepG2	MTT; flow cytometry	Western blot		^106^
**88** MS3227	MDM2	U-937, HCT 116, THP-1, MOLM-13, OCI-AML3, MOLT-4, HEL, MCF7, AML	Flow cytometry; CellTiter-Glo; Annexin V-PI	Western blot	*In vitro*: RT-qPCR	^107^
**89** (YX-02-030)	MDM2	DU4475, MCF7, MDA-MB-231, MDA-MB-436, MDA-MB-453, HCC-1143, HCC-1395, HCC-1937, TNBC tumour and normal breast tissue from adjuvant-treated patients	MTT; CellTiter-Glo; Annexin V, Caspase-3/7	Western blot	*In vitro*: homogeneous time resolved fluorescence; surface plasmon resonance; Alpha Screen; colony formation; mammosphere assay; chromatin immunoprecipitation; RT-qPCR; RNA-seq; metabolic stability *In vivo*: CD-1 mice (pharmacokinetics); female athymic nude mice (tumour xenograft studies with MDA-MB-231 or MDA-MB-436 cells (blood collecting, tissue H&E staining, apoptoti)	^112^
**90** 11a	MDM2	A549, HTC116, MCF7, HepG2	CCK-8; Annexin V-FITC	Western blot	*In vivo:* BALB/c nude female mice (tumour xenograft studies with A549 cells) (tumour volumes)	^104^
**91** (CM11)	VHL	HeLa, U2OS, HEK293		Western blot; mass spectrometry	*In vitro*: HRE-luciferase reporter assay; qRT -PCR; isothermal titration calorimetry; AlphaLISA assay	^19^
**92 (**14a)	CRBN	HeLa, HEK293		Western blot		^113^
**93 (**15a)	CRBN	MM.1S, U266, KMS27, LP-1, NCI-H929, OPM-2, K562, OCI-AML5, HEK293T	CellTiter-Glo	Western blot; mass spectrometry	*In vitro:* ubiquitination;	^103^
**94** (CRBN-6-5-5-VHL)	CRBN	MM.1S, K562	CellTiter-Glo	Western blot		^114^
**95** (TD-165)	CRBN	HEK293T, Jurkat, 786-O		Western blot; mass spectrometry	*In vitro*: permeability (the PAMPA Explorer Test System) *In vivo:* male ICR mice (pharmacokinetics)	^115^
**96** (ZXH-4-130) **97** (ZXH-4-137)	CRBN	MM.1S, MOLT-4, SK-N-DZ, HEK293T, Kelly	CellTiter-Glo	Western blot; mass spectrometry		^116^
**98** (TD-1092)	IAP	MCF7, HEK293T, MDA-MB-231, SKOV3, MDA-MB-157	CellTiter-Glo; FITC Annexin V	Western blot; ELISA; mass spectrometry	*In vitro*: Caspase 3/7 activity; RT-qPCR; TurboID assay; transwell migration and invasion assay	^109^
**99** (9)	IAP	HEK293T, MM.1S, HG3	CellTiter-Glo	Western blot; mass spectrometry		^117^
**100** (dTRIM24)	TRIM24	MV4;11, MOLM-13, OCI-AML, HL-60, NOMO-1, THP-1, 293FT, MCF7, KASUMI-1	Flow cytometry	Western blot; mass spectrometry	*In vitro*: Alpha Screen; chromatin immunoprecipitation with sequencing; VHL degron displacement assay	^108^
**101** (NJH-04-087)	KEAP1	OVCAR8, PATU-8988T, JeKo-1, Mino, MM.1S, KPC	CellTiter-Glo	Western blot; mass spectrometry	*In vitro:* RT-qPCR,	^118^
**102** (14)	KEAP1	HEK293T, HCA7, A459, BEAS-2B	Flow cytometry	Western blot; mass spectrometry	*In vitro*: RT-qPCR; ARE-luciferase reporter assay; permeability (the PAMPA Explorer Test System)	^110^

### PROTACs targeting MDM2

MDM2, which inhibits the tumour suppressor p53 and is overexpressed in a variety of human malignancies, is one clear target for cancer therapy. The high-resolution co-crystal structure revealed the interactions between the α-helical conformation of p53 and the deep hydrophobic cleft of MDM2^119^. It is anticipated that small compounds interfering with MDM2-p53 or reducing MDM2 activity will activate the p53 pathway, causing cancer cell-cycle arrest and death. Several small-molecule drugs, including nutlins, have entered human clinical trials as a consequence of years of intensive study and have shown anti-tumour benefits. However, two significant issues result from these clinical trials, acquired resistance due to p53 mutations and drug-related toxicities. Li et al. developed the first powerful MDM2 degraders using the CRBN ligase binders and the MDM2-p53 spirooxindole inhibitor MI-1061^105^. The carboxylic acid group in MI-1061 was used as the tethering site for the linkage with the CRBN ligands. The authors also demonstrated that a VHL-based system may not be suitable for effective MDM2 degraders. The most promising compound MD-224 (**85**) with lenalidomide inhibited cell proliferation at low nanomolar concentrations in a panel of human leukaemia cells. In RS4;11 cells, MI-1061 increased MDM2 protein levels in addition to the desired increase in the p53 protein. Transcriptional increase of *MDM2*, the cell cycle regulator gene *p21*, and the pro-apoptotic *PUMA* was detected, but not *TP53*, the gene encoding p53. The use of **85** also resulted in *MDM2* transcriptional gene upregulation, but MDM2 protein was substantially reduced. A single 25 mg/kg iv dose of **85** resulted in a time-dependent decrease in MDM2 protein levels in the RS4;11 xenograft model. Multiple intravenous doses of 8 mg/kg every second day demonstrated up to 50% tumour regression with no notable weight loss or other symptoms of toxicity. The same group discovered later MG-277, characterized by further structural simplifications of **85**. MG-277 was significantly less potent than **85** at causing MDM2 degradation and failed to activate WT p53. However, this compound could considerably suppress cell proliferation in both RS4;11 cancer cells and p53 mutant RS4;11/IRMI-2 cells. MG-277 behaved as a molecular glue rather than a PROTAC and it efficiently degraded the translation termination factor GSPT1^120^.

Using short alkynyl-chained linkers to couple a Nutlin-3 derivative RG7112 to lenalidomide, Tang and colleagues synthesized a new class of MDM2 degraders with compound 32 (**86**) characterized by the strong antiproliferative effect. **86** has one of the shortest and rigid linker (derived from the propargyl group) among all reported PROTACs. It induced efficient degradation of MDM2 at low nanomolar concentrations in RS4; 11 leukaemia cells carrying wild-type p53^111^. The same group designed a potential MDM2 degraders with ligands synthesized by a four-component Ugi reaction. The compound WB214 was shown to be the most effective anti-proliferative agent in a variety of leukaemia cell lines. But unexpectedly WB214 not only successfully triggered the degradation of MDM2, but also p53, acting as a molecular glue^121^.

In 2021, Qi et al. synthesized a number of degraders based on pomalidomide and ursolic acid (UA), a pentacyclic triterpenoid found in natural sources. Ursolic acid has a wide range of pharmaceutical properties, including anticancer, anti-inflammatory, antiviral, and antibacterial. PEG units of various lengths were used to couple UA via the carboxyl group with thalidomide. 1B (**87**) with a 3PEG linker appeared to be the most effective MDM2 degrader, which also showed significant cytotoxic effects in A549, Huh7, and HepG2 by increasing the levels of p21 and PUMA proteins. These results served as proof-of-concept for the development of UA-based PROTACs that target MDM2^106^.

Marcellino et al. choose AMG 232 as the MDM2 small molecule ligand scaffold and VHL as E3 recruited ligase to design MDM2-targeting PROTACs^107^. The authors demonstrated higher VHL expression in acute myeloid leukaemia cells (AMLs) as compared with other cancer subtypes and normal tissues. Therefore, using VHL for PROTAC design may provide higher selectivity towards leukemic cells against normal haematopoietic cells and non-haematopoietic tissues. MS3227 (**88**) bearing 8-carbon linker appeared to be the most effective MDM2 degrader. **88** activated the p53 pathway, stimulated transcription of downstream targets, and enhanced apoptosis in *TP53* WT leukaemia cell lines. **88** was also examined in primary AML samples and found to be active in samples with varying cytogenetic and mutational characteristics with selectivity towards leukemic blasts. The authors found that leukaemia cell lines were more sensitive to **88** than solid tumour lines, implying that AML cell function and viability are particularly dependent on this route. **88** increased the activity of other anti-leukemic drugs such as azacytidine, cytarabine, and venetoclax. **88** therapy, in particular, was demonstrated to downregulate MCL1, a known mediator of venetoclax resistance. A PROTAC-based strategy to MDM2 inhibition may provide a technique of enhancing therapeutic potential in AML.

Previous studies targeting MDM2 with heterobifunctional PROTACs 104^111^, mostly focused on acute lymphoblastic leukaemia. Adams et al. designed MDM2-targeted PROTAC, YX-02–030 (**89**) with RG7112 tethering VHL E3 ligase recruiting VH032 via a PEG linker and proved its efficacy in p53-inactivated TNBC^112^. Inactivation of p53 occurs frequently in TNBC which frequently develops *TP53* mutations, with nonsense mutations resulting in p53 protein loss and missense mutations impairing the function of the transcription factor. Compounds that prevent p53 from binding to MDM2 are ineffective in TNBC due to their high rates of p53 inactivation. Extensive research demonstrated that **89** effectively killed TNBC cells with three gain-of-function mutations (R280K, R248Q, and R175H) or deleted p53 in two-dimensional and three-dimensional culture models, patient explants, and tumour xenografts by activating the p53 family member TAp73. Normal human breast epithelial or stromal cells and CD34^+^ haematopoietic cells were not affected by **89**, and similarly, in mice, there was no toxicity detected in haematopoietic or other tissues, significantly reducing concerns about globally or locally activating wild-type p53 in normal tissues with MDM2 degradation. Notably, VHL levels are particularly low in platelets when compared to cancer cells, and therefore VHL-recruiting PROTACs could be advantageous in lowering the risk of patients developing thrombocytopenia.

A novel variation of the traditional PROTACs, homo-PROTACs, allows for the direct coupling of two identical E3 ligase ligands inducing just certain types of self-degradation. The advantage of homo-PROTACs over standard PROTACs is predicated on their inability to engage a second target, which may prevent the induction of toxicity and/or adverse effects. Although conventional hetero-PROTACs have numerous advantages over small-molecule inhibitors, in addition to binding POI, interaction with additional targets, generating toxicity and side effects may be observed. For example, PROTACs designed to degrade receptor tyrosine kinases were not able to elicit degradation of its consensus targets. Instead, the translation termination factor G1 to S phase transition 1 (GSPT1) was identified as a convergent off-target^122^. Homo-PROTACs have recently been reported to target MDM2^104^, VHL, and CRBN, revealing new chemical probes to achieve the selective degradation of E3 ligases and offering promising cancer intervention techniques.

He et al. combined two molecules of *cis*-biphenyl-substituted imidazoline compound, a derivative of classic MDM2 inhibitor nutlin-3, to develop the first MDM2-based homo-PROTACs. PROTAC 11a (**90**) successfully degraded MDM2 via the proteasome pathway and induced the upregulation of the p53 protein by binding to MDM2. **90** had the strongest antiproliferative effects causing apoptosis in A549 cells. However, **90** demonstrated limited permeability. It was also discovered that enantiomer 11a-1 had greater MDM2 degradation activity. Particularly, homo-PROTAC 11a-1 revealed a good PK/PD profile strongly suppressed tumour growth in A549 xenograft models without any obvious toxicity or negative effects^104^.

### PROTACs targeting VHL

In 2017 Maniaci et al. developed first homo-PROTACs for the auto-induced degradation of VHL, as a proof-of-concept. They synthesized three classes of PROTACs, by combining two different attachment point on the VHL032 ligand. The most efficient PROTAC, compound CM11 (**91**), was symmetrically linked via *tert*-leucine moiety of VH032. **91** avidly formed a 1:2 complex with VHL inducing proteasome activity and Cullin2 (a part of the CRL2^VHL^ complex) neddylation in different cell lines. Increased cellular activity of >1000-fold compared to the parent inhibitor VH032 was observed. The preferential degradation of long isoform pVHL30 at nanomolar concentrations over the short VHL isoform pVHL19 was unexpected and is an intriguing outcome of this study. As pVHL19, a preferential part of the CRL2^VHL^ complex, was not affected, **91** had no effect on hypoxia-inducible factor alpha (HIF-α) which is recognized by VHL^19^. This finding suggests that chemical degraders made from inhibitors that recruit more than one protein can show higher selectivity compared with the parent inhibitor independent of target engagement. However, due to the tumour suppressor role of VHL, the PROTAC-mediated degradation of VHL may promote tumorigenesis, necessitating further mechanistic research by the homo-VHL PROTAC probes to understand the cellular action.

### PROTACs targeting CRBN

Subsequently, the same Ciulli group extended their homo-PROTAC approach hypothesising that two different E3 ligases could be brought together using hetero-bifunctional PROTACs made of a ligand handle for one ligase and another handle for a different ligase. They designed several VHL-CRBN hetero PROTACs with the most active compound 14a (**92**). As a CRBN handle pomalidomide was chosen, linked with three different locations in VH032 ligand. They observed significant degradation of CRBN while no significant degradation of VHL, with any of the compounds tested when used at 1 µM concentration. However, at lower 10 nM concentration some compounds induced to some extend degradation of pVHL30. Thus, depending on the concentration used, preferential degradation of one ligase over the other can be induced. No degradation of short form pVHL19 was visible consistent with the activity of **91**^113^.

In 2018, homobifunctional PROTACs were developed with CRBN as both the hijacked degrader and the protein targeted for degradation. Two pomalidomide moieties were connected using linear linkers varying in length, hydrophobicity, and attachment position. All homo-PROTACs reduced IKZF1 protein levels in a dose-dependent manner. The compound 15a (**93**) with 2PEG linker was shown to be the most effective CRBN degrader with only minor impacts on the IKZF and no effect on CUL4A, a part of the CRL4^CRBN^ E3 ligase complex and casein kinase 1α, a CRBN neo-substrate. **93** induced CRBN ubiquitination and degradation by forming ternary complexes of a 2:1 stoichiometry with two CRBNs and one PROTAC, rather than by autoubiquitination or binding to another ubiquitin ligase^103^.

In the next year, Steinebach et al. designed a series of heterodimeric molecules, out of which each PROTAC tethered two different ligases, i.e. CRBN and VHL and similarly to **92**^113^ showed preferential degradation of CRBN over VHL^114^. Notably, CRBN-6–5-5-VHL (**94**) with 18 atom-long polyether linker had no influence on the degradation of the neo-substrates IKZF1 and IKZF3 and outperformed CRBN homo-PROTAC **93** in terms of CRBN depletion and avoiding neo-substrate degradation. These results demonstrated the desirable properties of the CRBN-VHL hetero-PROTACs as non-toxic, potent, and selective CRBN-depleting PROTACs. By blocking the induction of IKZF1 and IKZF3 degradation, **94** was able to protect or rescue MM.1S cells from IMiD toxicity. The broad application of **94** was proven further by measuring CRBN levels in several cell lines. Degradation of only one E3 ligase, CRBN, but not VHL could be due to an unrequited ubiquitin transfer and VHL may be protected from ubiquitination.

VHL-CRBN PROTACs developed by Kim et al. also induced the degradation of CRBN, but not VHL^115^. IKZF1 and IZKF3 were not affected as well. The most selective and potent TD-165 (**95**) was evaluated to explain the selectivity for CRBN degradation. Studies on protein degradation in cells overexpressing CRBN, VHL, or both E3 ligases demonstrated that relative protein levels did not affect protein degradation. After analyzing several CRBN deletion mutants, it was discovered that the disordered region of full-length CRBN is crucial for effective CRBN degradation. These findings thus provide a novel criterion for selecting targets for degradable proteins by indicating that the intrinsically disordered region of targeted proteins is necessary for effective proteolysis.

The Gray group applied a focused combinatorial library approach to synthesise two sets of CRBN–CRBN and CRBN–VHL PROTACs^116^. Hetero-PROTACs, ZXH-4–130 (**96**) and ZXH-4–137 (**97**) which differ from **95**^115^ by the atom (O vs. N) connecting pomalidomide with second ligand, were capable CRBN degraders in MM.1S, Kelly, HEK293T, SK-N-DZ, and MOLT-4 cell lines. The 11-carbon linker was the same, and the VHL ligand in **97** was a methylated version of the VHL ligand in **96**. CRBN was the only protein target of **96** and **97** that was significantly downregulated and other members of the CRBN E3 ligase complex, DDB1, CUL4A, and ROC1, were not affected. In comparison to **92** and **95**, **96** and **97** appeared to be more potent, while similar potency with **85** and **94** was demonstrated.

### PROTACs targeting IAP

Cellular IAP 1/2 (cIAP1/2) and X-linked IAPs (XIAPs) play a key role in immune signalling and cancer development. CRBN–IAP heterobifunctional PROTACs were developed very recently by Park et al.^109^ and Ng et al.^117^ Compound 1 was selected by the first group as an IAP binder tethering thalidomide with PEG or aliphatic chains^109^. TD1092 (**98**) containing octyl-3-azetidine carboxylic acid linker was superior in inducing cIAP2 and XIAP degradation in a CRBN-dependent manner and cIAP1 in a CRBN-independent manner in MCF7 cells. Besides all three IAPs, **98** induced the degradation of the melanoma inhibitor of IAP (ML-IAP) highly expressed in melanoma cell lines while the CRBN level was not changed. **98** outperformed IAP antagonists in terms of cytotoxicity and TNF signalling pathway inhibition. **98** inhibited innate immune response induced by TNFα and cancer cell migration and invasion, resulting in apoptotic cell death.

Ng et al. demonstrated the comprehensive evaluation of 32 hetero-PROTACs aiming at IAP degradation^117^. Two series were developed by connecting the IAP ligand to two VHL ligands with variously oriented exit vectors. Pomalidomide was incorporated into the third library. Eight linkers varying in length and chemical composition were introduced in each series. Maximum cIAP1, cIAP2, and XIAP degradation was achieved by IAP-VHL hetero-PROTAC 9 (**99**). The pan-IAP degrader **99** potently inhibited the growth of nine hematological cell lines including multiple myeloma, acute myeloid leukaemia, and diffuse large B-cell lymphoma. The authors also observed bidirectional degradation within IAP-VHL degraders but this effect was less significant for PROTACs with longer hydrophobic linkers. A reduction of pVHL30 and pVHL19 levels is in contrast with previously published CRBN-VHL hetero-PROTACs where no degradation of pVHL19 was observed^113^. Notably, an isoform-selective XIAP degradation was observed for IAP-CRBN hetero-PROTAC with the longest linker. This finding demonstrates that linker modifications can be used to tune IAP selectivity within the IAP-CRBN hetero-PROTAC series.

### PROTACs targeting TRIM24

A first bifunctional degrader of TRIM24 was reported by Gechijian et al.^107^ TRIM24 is a multidomain protein with RING domain that functions as an E3-ubiquitin ligase to regulate p53 protein levels, similar to MDM2. An approach to restoring p53 functions and causing tumour cells to undergo induced apoptosis may be provided by therapeutic targeting of the TRIM24 protein^123^. However, known TRIM24 inhibitors are unable to suppress the majority of cancer cells. TRIM24-targeting PROTAC dTRIM24 (**100**) was synthesized by fusing the selective dimethylbenzimidazolone TRIM24 bromodomain inhibitor IACS-9571 with a CRBN ligand. **100** and the TRIM24 ligand had comparable IC_50_ values but **100** more actively removed TRIM24 from chromatin having a remarkable impact on TRIM24 transcription across the entire genome. In 293FT cells, **100** specifically degraded TRIM24. The study did not, however, evaluated if VHL was also degraded.

### PROTACs targeting KEAP1

Recent development of KEAP1-CRBN PROTACs^109^^,^^110^, that preferentially degrade KEAP1 have expanded the list of heterobifunctional ligase degraders. The Kelch-like ECH-associated protein 1 (KEAP1), the cullin3-RING ligase family member, has the ability to ubiquitinate erythroid 2-related factor-2 (NRF2), a crucial transcription factor that controls the expression of antioxidant proteins. However, key cysteine residues in cysteine-rich protein KEAP1 are modified in response to the oxidative or electrophilic species, which hinders interaction with NRF2 and prevents its degradation. Therefore, NRF2 escapes from KEAP1-mediated degradation and translocates to the nucleus, where it induces the expression of antioxidant response element (ARE)-directed genes^124^.

Du et al. explored a large library of PROTACs with KEAP1 inhibitor KI-696 as a KEAP1-recruiting ligand^118^. Among KEAP1-based PROTACs a transcriptional and epigenetic regulator BRD4 and focal adhesion kinase (FAK) were identified as degradable targets. However, in contrast with VHL- and CRBN-based PROTACs, degradation of Bruton’s tyrosine kinase (BTK), epidermal growth factor receptor (EGFR), or cyclin-dependent kinase 4/6 were not achieved what suggests that KEAP1-based PROTACs may have a more constrained target space. Notably, by tethering KI-696 to a ligand of the CRBN E3 ligase through 9-atom linkers, two powerful KEAP1 degraders NJH-04–086 and NJH-04–087 were developed. Both compounds quickly degraded KEAP1, but they spared CRBN. However, NJH-04–086 was less selective than NJH-04–087 (**101**) as downregulation of some zinc finger proteins was also observed during proteomics profiling in OVCAR8 cells.

Chen et al. disclosed a series of KEAP1-CRBN PROTACs used as a mixture of diastereomers (*R*,*S*-at the 3-position of the propanoic acid) of KEAP1 inhibitor (compound 2)^110^.

The lead compound 14 (**102**) with a 7-atom linker and an amino-carbon linkage to thalidomide showed potent KEAP1 degradation with low nanomolar DC_50_ in HEK293T and BEAS-2B cell lines. Additionally, **102** boosted the expression of antioxidant proteins that are controlled by NRF2 and prevented cell death brought on by reactive oxygen species.

### Deubiquitination enzymes

Deubiquitination enzymes can trim the conjugated Ub molecule away from the target protein (DUBs). The human genome encodes ≈100 of DUBs, which are classified into seven subfamilies (Table 15). Six families of cysteine proteases include Ub-specific proteases (USPs), Ub C-terminal hydrolases (UCHs), ovarian tumour proteases (OTUs), Machado-Josephin domain-containing proteases (MJDs), MIU-containing novel DUB (MINDY), and zinc finger with UFM1-specific peptidase domain protein/C6orf113/ZUP1 (ZUFSP), are among the six subfamilies (cysteine-dependent proteases). The seventh family, Jab1/MPN domain-associated metallopeptidase (JAMMs), is made up of zinc-dependent metalloproteinases^125^.

**Table 15.** Categorisation of deubiquitination enzymes.

DUBs						
USPs	UCHs	OTUs	MJDs	MINDY	ZUFSP	JAMMs
USP1-USP6, **USP7**, USP8-USP22, USP24- USP54, CYLD	UCL1, UCL3, UCL5, BAP1	OTUB1, OTUB2, OTUD1, OTUD3, OTUD4, OTUD5, OTUD6, OTUD7, OTULIN, YOD1, STAMBP, A20, Cezanne, Cezanne2, TRABID, VCPIP1, FAM105A	ATXN3, ATXN3L, JOSD1, JOSD2, JOSD3	MINDY1, MINDY2, MINDY3, MINDY4	ZUFSP	STAMBP, STAMBPL1, BRCC36, COPS5, COPS6, PSMD7, PSMD14, PFPF8, EIF3F, EIF3H, MPMD, MYSM1

Enzymes for which PROTACs have been developed are marked in bold

DUBs recognize and cleave the isopeptide bonds that connect ubiquitin to proteins, and they play an important role in protein stability, homeostasis, and signalling in cells. DUBs emerged as promising targets that, if successfully inhibited or depleted, could supplement the drug inventory for a variety of diseases, particularly cancer. USP7 is a DUB that has been linked to a number of tumour-suppressor genes and proto-oncogenes, indicating its importance in tumour biology and progression. USP7 is expressed at high levels in many cancer tissues and is frequently associated with poor prognosis and metastasis. It is a key regulator of the tumour suppressor p53, either directly or indirectly, by stabilizing MDM2^126^.

### PROTACs targeting deubiquitination enzymes

**Figure 12.**
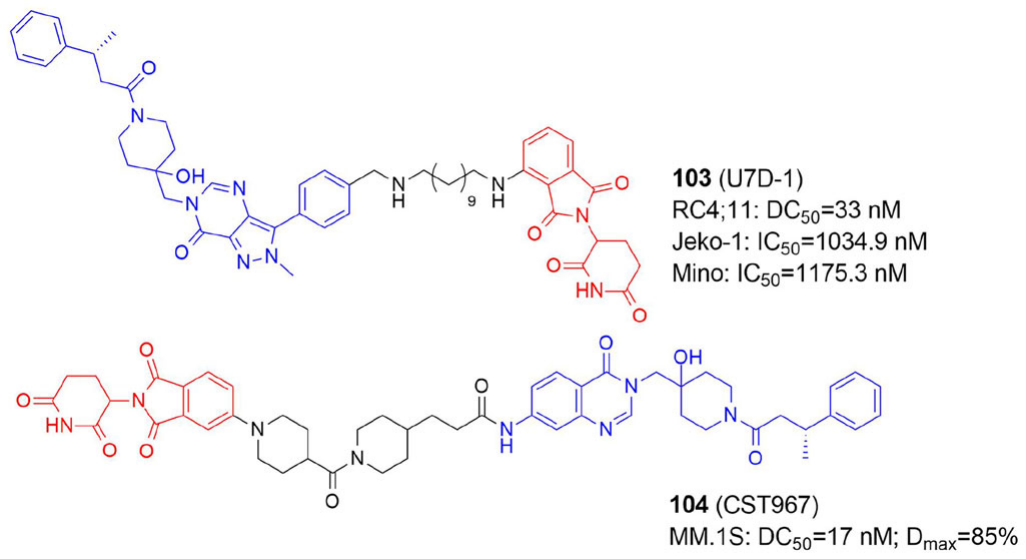
Structures and activities of PROTACs targeting deubiquitination enzymes.

Pei et al. developed U7D-1 (**103**) (Figure 12, Tables 16 and 17), the first potent and highly selective USP7 degrader, by choosing compound 4 which occupies an allosteric binding site of USP7. **103** inhibited cell growth in wild-type p53 cell lines, similar to USP7 inhibitors, and hampered cell growth in p53 mutant cell lines while conventional inhibitors had no effect. Cell growth inhibition caused by USP7 inhibition was p53-dependent and occurred via the regulation of the MDM2-P53-P21 signalling pathway^127^.

**Table 16.** Components and structural details of DUBs-targeting PROTACs.

PROTAC	Target	# of analogs	Original inhibitor	E3 ligase ligands	Type of linker (# of analogs)	Comments	Ref
**103 (**U7D-1)	USP7	7	Compound 4	CRBN	Alk (5) PEG (1) PEG-triazole (1)		^127^
**104 (**CST967)	USP7	17	XL188	CRBN	Alk (12) PEG-Alk (5)	Moderate lipophilicity (logD 2.5) was beneficial for degrader activity. Removing one or two carbon atoms in the linker increased rigidity but negatively affected the degradation potency.	^128^

**Table 17.** Experimental methods and cell models applied in development of DUBs-targeting PROTACs.

PROTAC	Target	Cell lines	Cell proliferation/cell cycle/apoptosis	Target degradation	Other assays	Ref
**103 (**U7D-1)	USP7	MV4;11, CCRF-CEM, Jeko-1, Reh, MOLT4, RS4;11, RPMI8226, OCI-ly10, SU-DHL-6, Mino	PMS-MTS	Western blot; mass spectrometry	*In vitro*: USP7 enzyme activity assay; RT-qPCR; RNA-seq	^127^
**104 (**CST967)	USP7	MM.1S, A549, LNCAP	CellTiter-Glo	Western blot; mass spectrometry	*In vitro*: USP7 enzyme activity assay	^128^

In the same year another series of PROTACs based on USP7 inhibitor scaffold was reported by Murgai et al. CST967 (**104**) appeared to be a highly potent degrader with growth-inhibitory activity in USP7-dependent cancer cells^128^.

## Summary and future perspective

**Figure 13.**
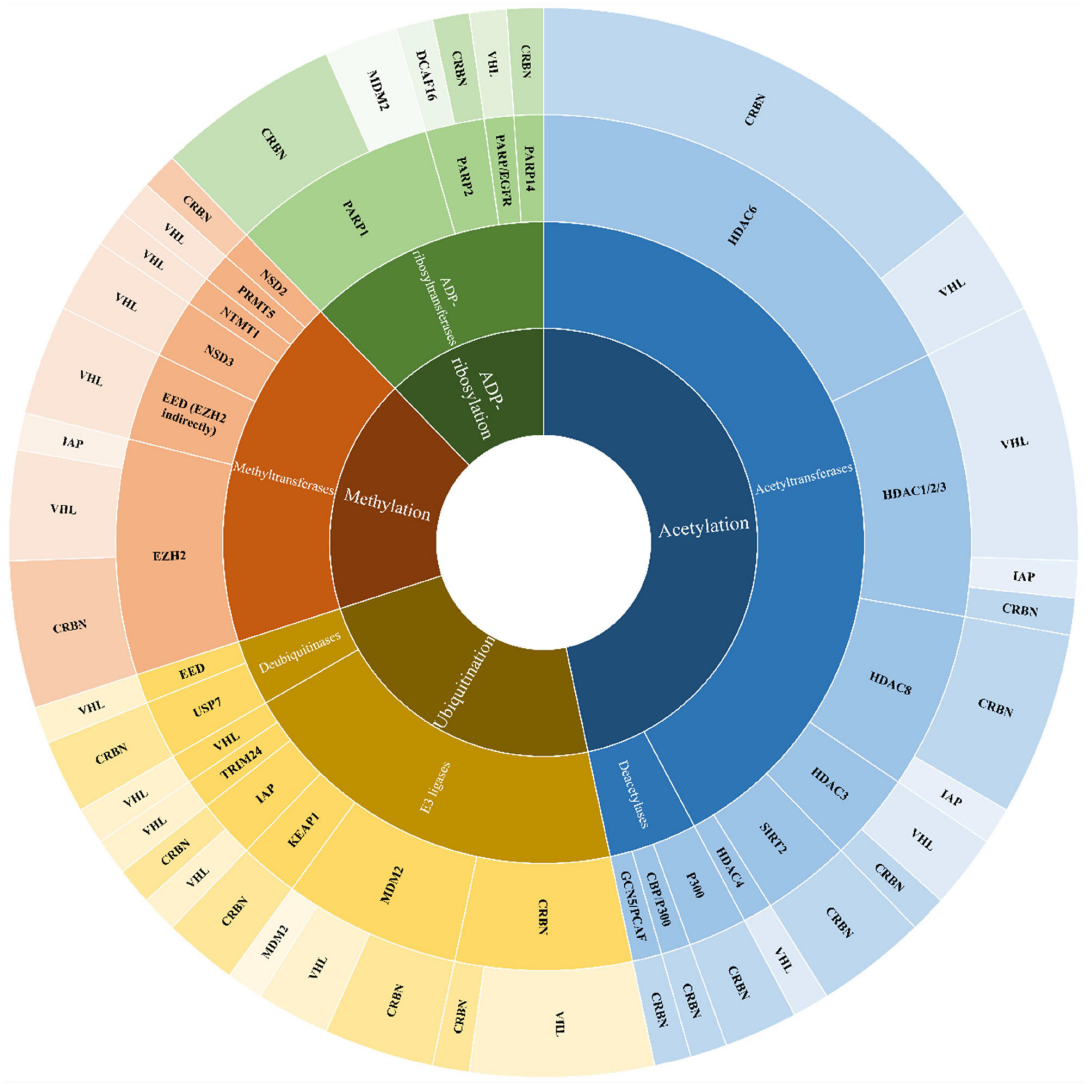
The pie chart summarising the privileged E3 ligase ligands depending on the class of POIs. The chart was prepared according to the number of the most active PROTACs listed in Tables presenting components and structural details of PROTACs.

PROTAC field is very advanced in targeting kinases, while the use of this technology for enzymes posttranslationally modifying lysine and arginine residues started to be developed only in 2018. Still, it already shows advanced development, addressing selectivity and discovering the phenomenon of collateral degradation. As we wanted to facilitate the lengthy experimental endeavours directed towards developing and optimisation of the structure of PROTAC, we brought together in clear, tabularised form all the analogs synthesized, showing the structural diversity of linkers and E3 ligase ligands originally employed, demonstrating the preferences (or their lack) for certain targets. The pie chart below is summarising the privileged E3 ligase ligands depending on the class of POIs (Figure 13). The most visible preference could be observed for HDACs, the group of enzymes constituting almost half of this chart, for which CRBN-based PROTACs turned out the most active, while only HDAC1/2/3 VHL is taking the lead role. Interestingly, the preferred localisation of HDAC6 and HDAC8 is the cytoplasm, while HDAC1/2/3 are localised also in the nucleus. However, no such correlation in the localisation of E3 ligases has been reported yet^129^. Overall, since E3 ligases are regarded as housekeeping enzymes, they are highly expressed in the majority of tissues with a limited except for a few examples, e.g. in platelets, the expression of VHL and CRBN is low^130^. Notably, most tissues express high levels of the most frequently used E3 ligases including CRBN and VHL. Broadly expressed E3 ligases include additional enzymes discussed above like cIAP, MDM2, KEAP1, DCAF16^131^.

Additionally, we recapitulated the methods currently used for PROTACs studies, both fundamental and advanced, as the community is still missing validation standards, and heavily depends on Western blotting, which is a relatively insensitive method and of low throughput. It could accelerate the choice between different tools available and include the appropriate control experiments.

Within this class of PROTACs, a phenomenon of collateral degradation (sometimes called bystander effect) of the proteins forming complexes with the POI targeted with PROTAC, has been reported for the first time. Large multiprotein complexes have historically been thought to be “undruggable” because the inhibition of one subunit may not have a complete negative impact on the function of the complex. It adds another dimension to the field: inhibitors lower the enzymatic activity of the targeted protein, PROTACs remove the protein, therefore its other functions (e.g. scaffolding, chaperone-like, etc.) are also reduced. Multiple complex members can be degraded by “bystander” ubiquitination or alternately, the complex may become unstable due to the degradation of one subunit, which may also cause the remaining complex subunits to degrade as a result of the natural protein quality control system. PROTACs leading to a deconstruction of the whole complex may assure that no residual or surrogate activity of other proteins will restore the particular function. Interestingly, such an effect was mainly observed for PRC2 and other complexes of EZH2 and EED proteins, and only a few reports show that it can characterise HDAC degraders (**42**–**44**: CoREST degradation), lysine methyl transferase NSD3 (**65** MYC degradation), or arginine methyltransferase PRMT5 (**68** evoked the loss of WDR77, a PRMT5 binding partner). Not all studies cited here were addressing this question, therefore it cannot be excluded that more events of complex degradation could be identified.

More common examples of the synergistic effect result either from purposeful co-application of PROTAC with a drug/inhibitor targeting certain enzymes or from the degradation of neo-substrates of E3 ligase CRBN, IKZF1/3. Another way is to use dual PROTACs, consisting of two warheads targeting two enzymes. Up to now, only one such example, **84**, targeting two enzymes, PARP and EGFR, has been reported for this class. In some cases, the combination of PROTAC and inhibitor increases the therapeutic effect. As far as co-degradation of CRBN neo-substrates is concerned, for **31** and **67**, degradation of POI together with IKZF1/3 improved the therapeutic outcome, making it a superior strategy. However, in some cases, such a phenomenon is not observed, e.g. for **94**–**97**. It is very individual if such a phenomenon is desirable or not since Ikaros degradation might be associated with potential toxicity. Based on the examples given, it is clear that studies aiming to answer how new PROTAC interacts with CRBN neo-substrates, should be a common practice.

The common topic in PROTAC development is the gain of selectivity upon transformation from inhibitor into PROTAC. That is also observed within the described class of analogs, with the most pronounced example of pan-HDAC inhibitor vorinostat, which, according to multiple reports, upon modification with linker and E3 ligase ligand, turned into less promiscuous PROTAC, mainly targeting HDAC6. Interestingly, such gain of selectivity could result from minor changes of PROTAC constituents, either E3 ligase ligand, warhead targeting POI, or linker, leading to a preference for one over the other target (e.g. HDAC, PARP1/2, E3 ligase in hetero-PROTACs). An interesting example constitutes PARP-directed PROTACs: change of solely POI targeting ligand (**75**, **76**) or E3 ligase ligand (**81**, **82**) was changing the selectivity between PARP1 and PARP2 targets, which is essential for this pair of proteins, as their dual deficiency promotes tumour growth in T cells. For hetero-PROTACs designed to target E3 ligases, major differences in the E3 ligase they target were observed upon change of the second E3 ligase ligand, but also adjustment of the linker could change the PROTAC from bidirectional into selectively targeting analog. On the other hand, there are examples where degradation of certain proteins, e.g. VHL by homo- and hetero-PROTACs, may also lead to undesirable effects of promoting tumorigenesis. Such examples lead to another question for the field if all proteins are good targets for this type of therapeutic modality.

Together, PROTACs emerge as promising therapeutics enabling target-specific degradation and will be for sure valuable strategies in developing future PTM modification enzymes and PTM protein isoform‐specific degraders for therapeutic applications. The development of more and more PROTACs for enzymes introducing or removing PTMs is anticipated to occur in the upcoming years, along with advancements in the chemical tools created by these ground-breaking studies. We believe that this work can guide future efforts towards developing optimised PROTAC analogs, based on the already existing data about certain targets.

## Data Availability

Data sharing is not applicable to this article as no new data were created or analysed in this study.
